# Photoswitches beyond azobenzene: a beginner’s guide

**DOI:** 10.3762/bjoc.21.143

**Published:** 2025-09-08

**Authors:** Michela Marcon, Christoph Haag, Burkhard König

**Affiliations:** 1 Institute of Organic Chemistry, University of Regensburg, Universitätsstr. 31, 93053 Regensburg, Germany

**Keywords:** photoswitches, photoswitch properties, synthesis of photoswitches, switching mechanisms, tutorial review

## Abstract

Approaching the vast, colourful world of photoswitches from a different field of study or as an undergraduate student may be overwhelming: azobenzene is undoubtedly the most famous due to its easy synthesis and the extensively studied properties. However, there are several photoswitch classes beyond azobenzene with interesting properties that can be tailored to meet one’s needs. In this tutorial review, we aim to explain the important terminology and discuss the synthesis, switching mechanisms, and properties of seven interesting photoswitch classes, namely azoheteroarenes, diazocines, indigoid photoswitches, arylhydrazones, diarylethenes, fulgides, and spiropyrans.

## Introduction

### Photophysical properties and switching mechanism

#### Key learning points

- Switching mechanisms and the change in properties.
- Overview of the major classes of photoswitches beyond azobenzenes.
- Main synthetic pathways for their preparation.

**Figure 1:**
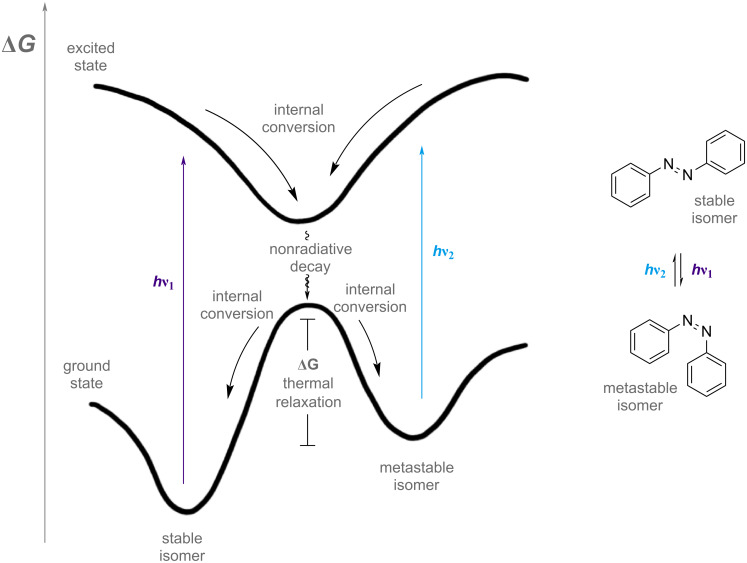
Energy diagram of a two-state photoswitch. Figure 1 was redrawn from [2].


Before introducing the different photoswitch classes in this review, we familiarise ourselves with different photophysical properties. A photochromic molecule can switch reversibly between a stable and a metastable state, which are shown in an energetic profile as two local minima (Figure 1). Shining light will bring the molecule to a generic excited state (which is different for different photoswitch classes and substitution patterns and will not be treated in detail), which can then relax back to the ground state in either of the two wells. In cases where the two absorption spectra of the two isomers do not overlap, only one isomer is excited by the irradiation wavelength, enriching the other isomer until an equilibrium is reached. This equilibrium is called a *photostationary state* (PSS) [1]. The isomeric distribution at equilibrium can also be called *photostationary distribution* (PSD). It must be noted, though, that in literature both PSS and PSD are used to indicate the photostationary distribution [2]. We will use the PSS notation throughout the review. Often, the two absorption spectra overlap, and both isomers are excited to different extents at the same time. The extent of excitation depends on the *molar extinction* ε of each isomer. It is the absorbance *A* normalised by the concentration *c* and the path length *l*, calculated according to the Beer–Lambert law:

[1] $$\varepsilon =\frac{A}{c\cdot l}$$

**Figure 2:**
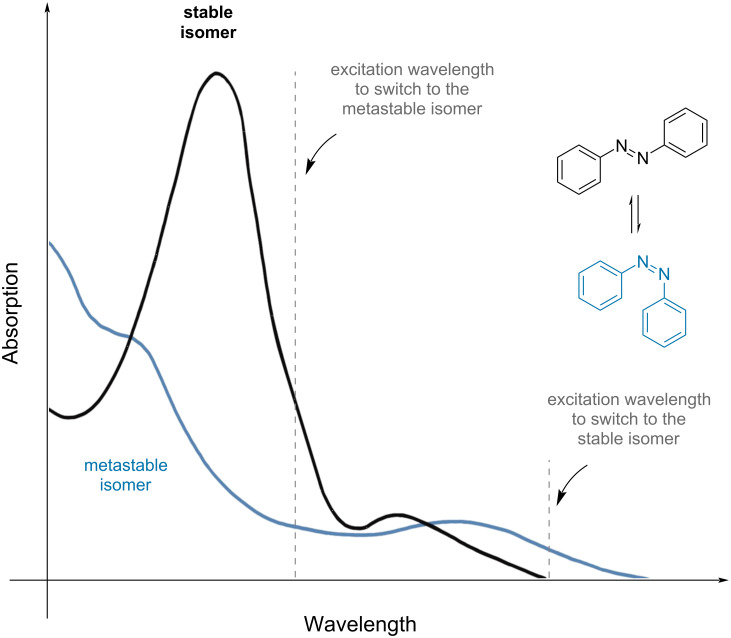
Example of the absorption spectra of the isomers of a photoswitch with most efficient irradiation wavelengths to obtain the highest PSS.

For this reason, careful choice of the excitation wavelength is important, as it affects how well one isomer can be switched to another. To achieve the best PSS, a wavelength with minimum spectral overlap should be chosen, which may not correspond to the maximum absorption wavelength (Figure 2) [3]. *Positive photochromism* is observed when the stable isomer has a maximum absorption at the lower wavelength (i.e., UV), and it is switched to one that absorbs at a higher wavelength (i.e., blue). The opposite phenomenon is called *negative* or *inverse photochromism* [3]. A shift towards higher energies is called *hypsochromic* or blue shift, while a shift towards lower energies is called *bathochromic* or red shift.

When working with a photoswitch, several factors must be considered: substituents, temperature, and solvent strongly affect the photophysical properties. For instance, the presence of electron-withdrawing (for instance -Cl, -Br, -CN, -NO_2_) and electron-donating groups (-alkyl, -OR, -NR_2_), which are directly conjugated with the chromophore structure, will influence its absorption spectrum and stability [4]. The solvent will also affect the properties, as it can stabilise or destabilise either the ground or the excited state, resulting in a red- or blue shift of the spectra. The *thermal half-life* (*t*_1/2_) measures the time within 50% of the metastable isomer is thermally converted to the stable one [3]. It depends on the photoswitch class, solvent, substitution pattern, and temperature. For some photoswitch classes, proton exchange and intramolecular hydrogen bonds are known to accelerate the thermal relaxation of the metastable isomer [5–7], thus they are also important factors to consider when choosing the solvent and the concentration of the solution. The half-life can be extrapolated by monitoring the photoswitch in the metastable form over time by various spectroscopic methods (UV–vis, NMR) in the absence of light. Apart from some special cases where more competing mechanisms are operating [5], the thermal isomerisation typically is a first-order decay:


[2] $$\begin{array}{l} I=I_{0}e^{-kt}\\ t_{1/2}=\frac{\ln2}{k} \end{array}$$

Where *I* is the monitored signal at the time *t*, *I*_0_ the initial signal, and *k* the kinetic constant. Once *k* is known, the thermal half-life *t*_1/2_ can be easily calculated. According to the thermal half-life, photoswitches are usually classified in *P-type* (thermally stable, with long half-life) and *T-type* (thermally unstable) [3]. The q*uantum yield* Φ measures the efficiency of the switching, and it relates the number of photons absorbed *n*_x_ to the number of irradiated photons *n*_tot_:


[3] $$\Phi =\frac{n_{x}}{n_{\text{tot}}}$$

Quantum yields vary in the range 0 ≤ Φ ≤ 1 and can be calculated by spectroscopic methods [8]. The calculation, however, is not trivial because irradiation can trigger various photochemical events. For instance, if the spectra of the two isomers strongly overlap, both forward and backward isomerisation can occur simultaneously. Moreover, some unwanted photochemical side reactions Φ_S_ can occur, as shown in the Equation 4:


[4] $$B'\overset{\Phi_{S}}{\leftarrow }A\overset{{h\nu }_{1},\Phi_{A\to B}}{\underset{{h\nu }_{2},\Phi_{B\to A}}{⇄}}B$$

*A* is one state of the photoswitch, *B* the second state, and *B’* a generic degradation product. *Fatigue resistance* measures how many times the photoswitch can be switched before it is degraded by side reactions. It is typically reported as *cyclability Z_50_*, which “is the number of cycles required to reduce the initial absorbance at a specific wavelength by 50%” [3]. The quantum yield Φ_S_ of these side reactions determines the resistance to fatigue of the photoswitch. Examples of photogenerated side reactions can be oxidation or irreversible rearrangements.

**Scheme 1:**
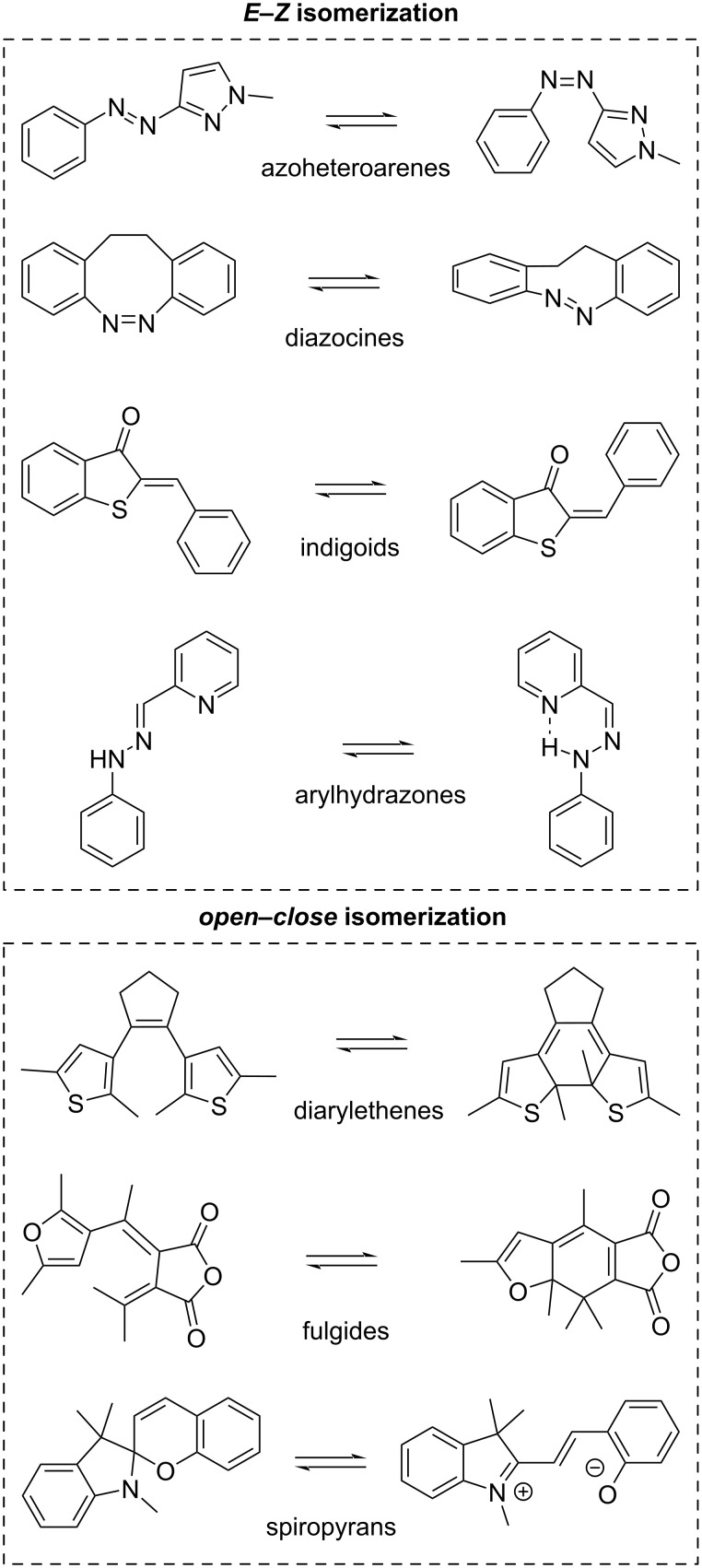
Photoswitch classes described in this review.

In the following sections, seven classes of photoswitches beyond the classic azobenzene are introduced and discussed (Scheme 1). Each of them shows unique photophysical behaviour and has individual features that may render them more suitable for applications than azobenzenes. This review, particularly for those new to the field, aims to summarise the classes of azoheteroarenes, diazocines, indigoids, arylhydrazones, diarylethenes, fulgides, and spiropyrans concerning their photophysical properties and tuneability while also outlining the most common synthetic methods for these compounds. Additionally, each chapter presents exemplary applications of the illustrated photoswitch classes, highlighting their practical relevance while inspiring the reader with new ideas in this field. For a more detailed view on photoswitches, such as in-depth analysis of photoswitching processes [9], their application into smart materials and biological systems [10], and the application in aqueous environments [2], we refer the interested reader to further reviews on these topics.

## Review

### Azoheteroarenes

**Figure 3:**
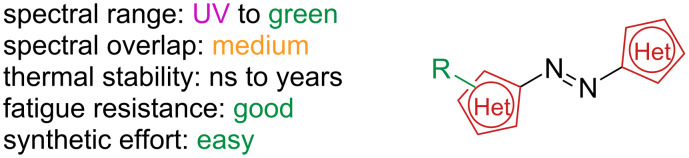
Azoheteroarenes.

Azoheteroarenes are azoswitches of which at least one aromatic ring is a heteroarene (Figure 3).

**Scheme 2:**
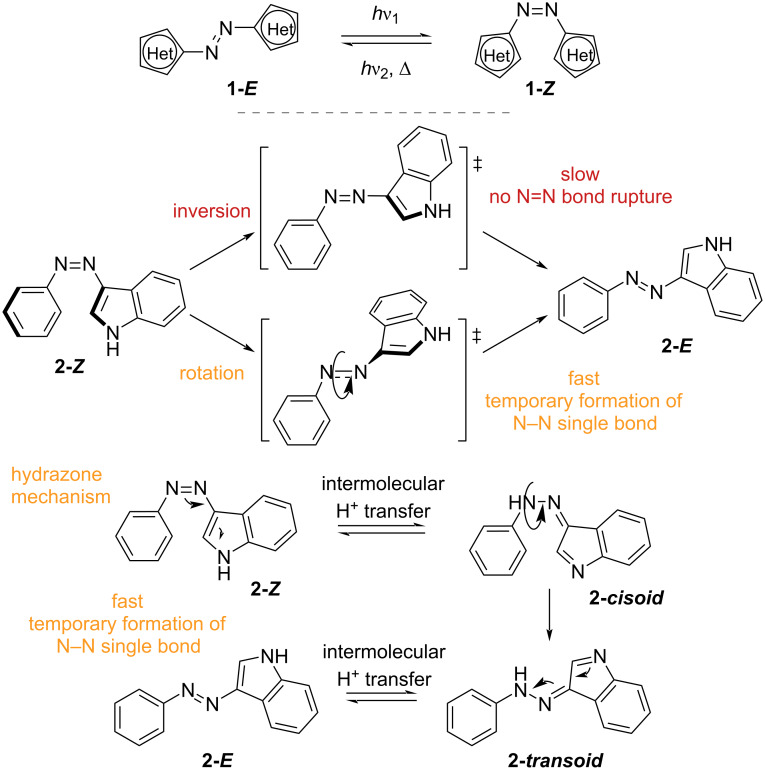
*E–Z* Isomerisation (top) and mechanisms of thermal *Z–E* isomerisation (bottom).

The variety of heterocycles and substitution patterns that can be used gives rise to a considerable number of variants covering all the UV and visible spectrum and with thermal *Z*-isomer lifetimes that span from picoseconds to several years. The presence of heteroatoms also introduces H-bond donors and acceptors and metal coordination sites [11]. Like azobenzenes, *E–Z* isomerisation of azoheteroarenes can be triggered by light irradiation, while *Z–E* isomerisation proceeds through irradiation with a different wavelength (P-type) or through thermal back isomerisation (T-type). As a general rule, the absorption can be red-shifted by the choice of electron-donating rings (pyrrole, thiophene), while electron-withdrawing rings (pyridine, pyrimidine, pyrazole, imidazole, thiazole) give the opposite effect [12]. Absorption red-shift is usually correlated to the shortening of the thermal lifetime [13]. To understand and to predict the thermal lifetime of these photoswitches, it is important to have a closer look at the thermal *Z*–*E* isomerisation, for which three main mechanisms have been found to take place: inversion (usually associated with longer lifetimes), rotation (usually associated to shorter lifetimes) and tautomerism (fast), shown in Scheme 2. The tendency between inversion and rotation depends on the degree of single-bond character of the N=N azo bond as well as the geometry of the *Z*-isomer [14]. Hydrazone tautomerism is an intermolecular process that depends not only on the choice of aryl rings and substituents but also on concentration, solvent polarity, and the presence of proton sources [5,15]. In conclusion, the choice of aryl rings, substituents, and substitution position are all crucial to determine the photophysical properties of this photoswitch class. The vast possibility of combinations and the straightforward syntheses make these compounds extremely attractive, however, somewhat difficult to strictly categorise: among the azoheteroarenes we find mainly nitrogen-based heterocycles such as pyrazole, pyrrole, pyridine, and indole, alongside with less common oxygen and sulphur-based heterocycles such as thiophene, thiazole, oxazole. At the end of the section, we will also briefly discuss heteroarylimines which, although not being azo-switches, resemble the geometry of azo-compounds and possess similar photophysical properties.

**Scheme 3:**
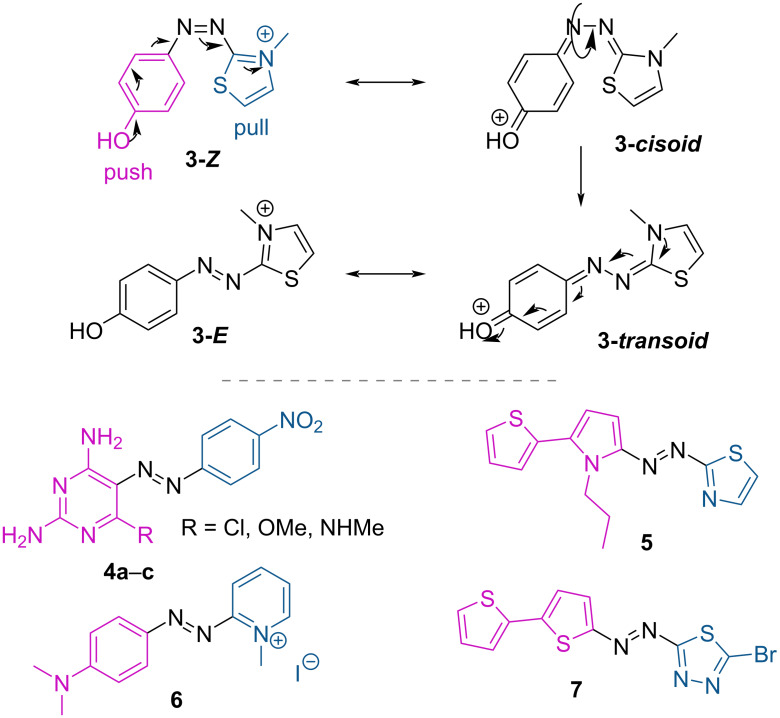
Rotation mechanism favoured by the electron displacement in push–pull systems. Selected examples of push–pull azoheteroarenes [17,20,24–25,27].

Push–pull systems, where a combination of an electron-donating group on one side of the azo bond (push) and an electron-withdrawing one on the other side (pull) decreases the double bond character of the N=N motif, are characterised by rotational thermal relaxation (Scheme 3) [16]. Due to the strong electron conjugation, these compounds usually show much shorter thermal lifetime, alongside with strong bathochromic shifts [17–26]. They are useful when fast responsive T-type photoswitches are needed. Moreover, some compounds show interesting non-linear optical properties, switching with two-photon absorption of near-IR wavelengths [3,27]. This could open the way to laser-storage devices and to application in biological systems, where longer wavelengths are preferred due to improved tissue penetration [28].

**Figure 4:**
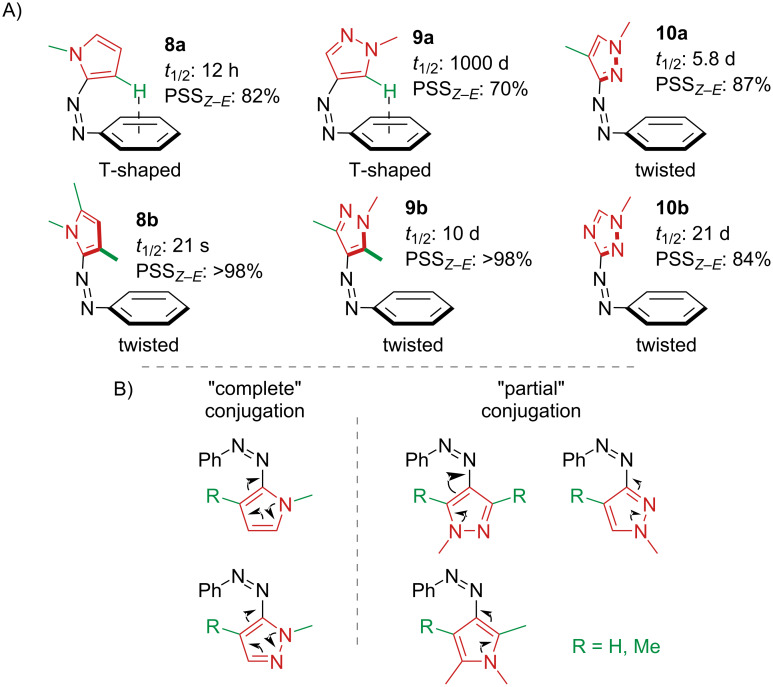
A) T-shaped and twisted *Z*-isomers determine the thermal stability and the *Z–E-*PSS (selected examples) [14,29]. B) Complete vs partial conjugation predicts the thermal stability.

When the push–pull character is not so strong as in the previous examples, a deeper analysis is usually needed to predict the photophysical properties of the azoheteroarenes. While the *Z*-isomer of 6-membered rings is always in a *twisted* conformation, it was discovered in the case of 5-membered rings that also a *T-shaped* conformation could be achieved in virtue of the reduced steric clash [29]. A systematic study on different azoheteroarenes with 5-membered rings demonstrated that the shape of the *Z*-isomer was responsible for the thermal lifetime of the photoswitch and for the PSS of the photoinduced *Z–E* isomerisation (Figure 4A) [14]. In case of T-shaped **8a** and **9a** the *Z*-isomer was more thermally stable thanks to the weak interaction between the hydrogen and the π-system. However, the high symmetry weakens the intensity of the nπ* absorption band, which is the excitation band for the *Z–E* isomerisation, resulting in a lower *E*-PSS. Basic nitrogen in “*ortho”* position (according to the nomenclature in the original paper) [14] also gives slightly twisted *Z*-isomers **10a** and **10b**. Moreover, a rough approximation of the thermal lifetime could be made considering the nature and connectivity of the heterocycle as either “complete” or “partial” conjugation (Figure 4B). The “complete” conjugation implies shorter lifetimes, due to the ability to rearrange the electrons more efficiently, thus showing a stronger push character.

**Figure 5:**
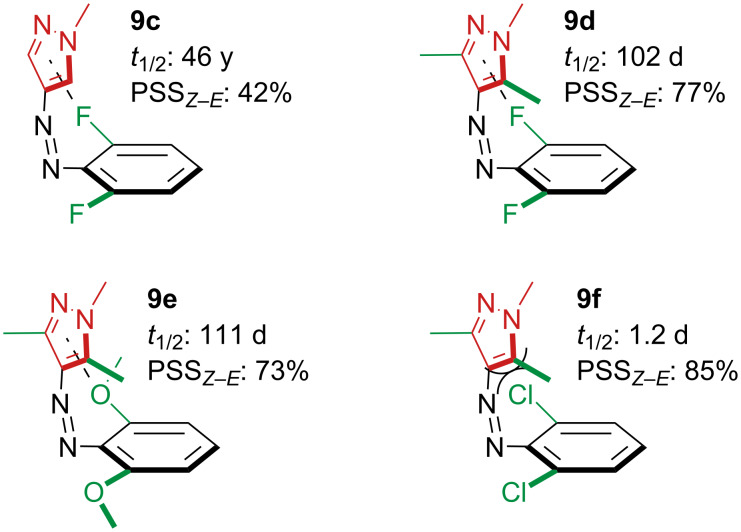
Effect of di-*ortho*-substitution on thermal half-life and PSS.

A follow-up study showed that di-*ortho*-fluorination (**9c** and **9d**) and di-*ortho*-methoxylation (**9e**) of the phenyl moiety of 4-phenylazopyrazoles notably increased the half-lives with respect to the unsubstituted ones, probably a result of favourable weak interactions between the pyrazole ring and the *ortho*-substituents in the *Z*-form [30]. Despite the high intensity of the nπ* absorption band, the absorption spectra of the two isomers are strongly overlapped, resulting in a less efficient switching. Conversely, di-*ortho*-chlorinated **9f** has lower thermal stability because of the steric clash with the bulky chlorine substituents (Figure 5).

**Figure 6:**
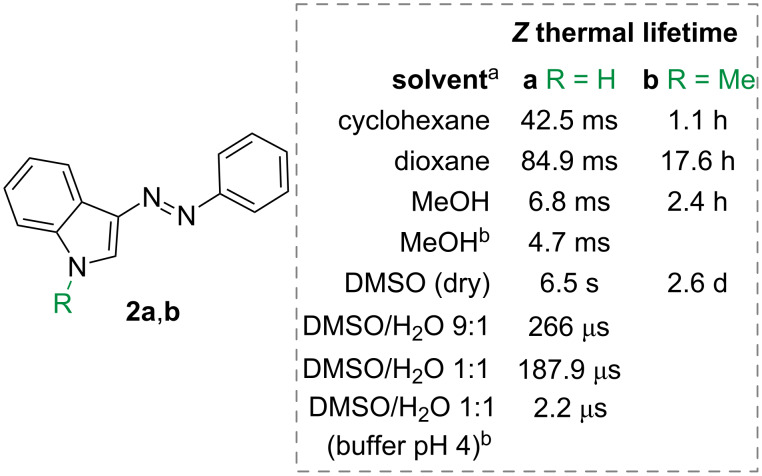
Selected thermal lifetimes of azoindoles in different solvents and concentrations. ^a^Concentration of photoswitch is 50 µM. ^b^Concentration of photoswitch is 75 µM [5].

An interesting study shows how the *Z*-isomer thermal lifetime changes between two phenylazoindole photoswitches (Figure 6) [5]. *N*-Methylation of indole **2b** increases the lifetime due to a preference for the inversion with respect to the rotation mechanism. Interestingly, the isomerisation of the non-methylated **2a** is also strongly influenced by protic solvents, by the pH of the solution, and by the concentration of the photoswitch in solution: when MeOH or water are present, the lifetimes drop significantly, and particularly at pH 4, as consequence of more favourable intermolecular proton transfer. Calculations show that water molecules bridging between two *Z*-isomers favour the formation of hydrazone and thus rapid conversion to the *E*-isomer.

A follow-up study calculated the activation energies of inversion, rotation, and tautomerisation of a number of previously reported and newly synthesised heteroaryl azo-switches, correlating the preferred mechanism of thermal back isomerisation to the reported lifetimes with great accuracy [7]. In summary, the more extended the conjugation of the heterocycle with the N=N bond, the more feasible the inversion mechanism (which does not involve the rupture of the double N=N bond). The less conjugated switches, however, would undergo rotation around the (instant) single bond N–N. The hydrazone pathway in protic solvents could be ruled out in cases when the energy of the *s-cis*-hydrazone was superior to the energy of the other two transition states. For further substituent effects on azoheteroarenes, we refer the readers to the following reports by Venkataramani, Corminboeuf, Samanta, and König [6,31–35].

**Figure 7:**
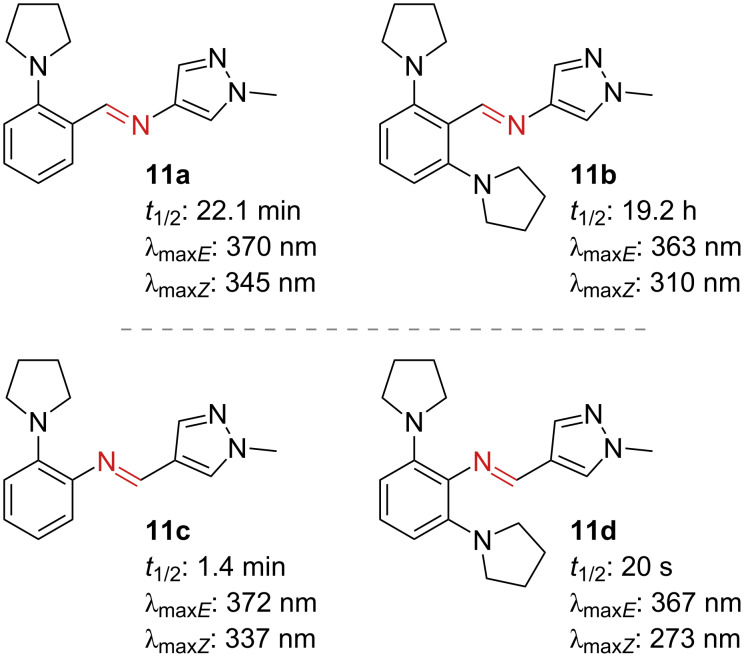
Aryliminopyrazoles: *N*-pyrazoles (top) and *N*-phenyl (bottom).

Although they do not possess an azo bond, heterocyclic imines are worth mentioning in this chapter due to their similarity to the azo-compounds and their straightforward synthesis. Substitution of the azo bond with an imine bond gives rise to heterocyclic imines [36–38]. The *Z*-isomers of the unsubstituted derivatives adapt different conformations: T-shaped for the *N*-phenyl and twisted for the *N*-pyrazoles. The half-lives follow the same trends already discussed beforehand. In contrast with the azopyrazoles, mono- and di-*ortho*-amination of the aryl ring of **11a** and **11b** yield iminopyrazoles with longer half-lives and negative photochromism (Figure 7, top). This unusual behaviour can be explained by the absence of adjacent non-bonding lone pairs in the imine bond [37]. Conversely, in the case of *N-*phenyl derivatives **11c** and **11d** (Figure 7, bottom), the thermal lifetimes drop significantly [38]. It is thus crucial to take into account the asymmetric nature of the imine bond and the steric hindrance of the substituents in the design of these photoswitches. For a detailed analysis of the structure–property relationship of these compounds we direct to recent literature [37–38].

#### Synthesis

**Scheme 4:**
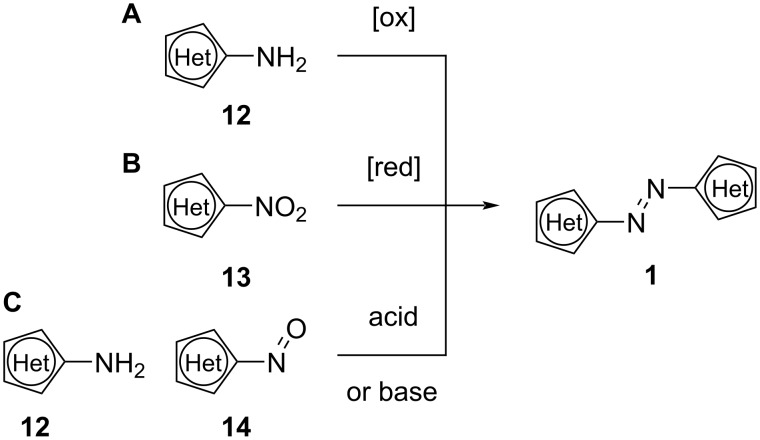
Synthesis of symmetrical heteroarenes through oxidation (A), reduction (B), and the Bayer–Mills reaction (C), also suitable for asymmetrical heteroarenes.

Many synthetic approaches have been developed for the synthesis of azoheteroarenes. Typically, synthesising this kind of photoswitches is rather straightforward and one can usually obtain azoheteroarenes with low synthetic effort. Symmetrical compounds can be synthesised through oxidation of aminoheteroarenes **12** (Scheme 4A) or reduction of nitroheteroarenes **13** (B). Bayer–Mills coupling (Scheme 4C) is suitable for both symmetric and asymmetric targets, usually in acidic conditions. Basic conditions [14] are more effective with very electron-poor aromatic amines.

**Scheme 5:**
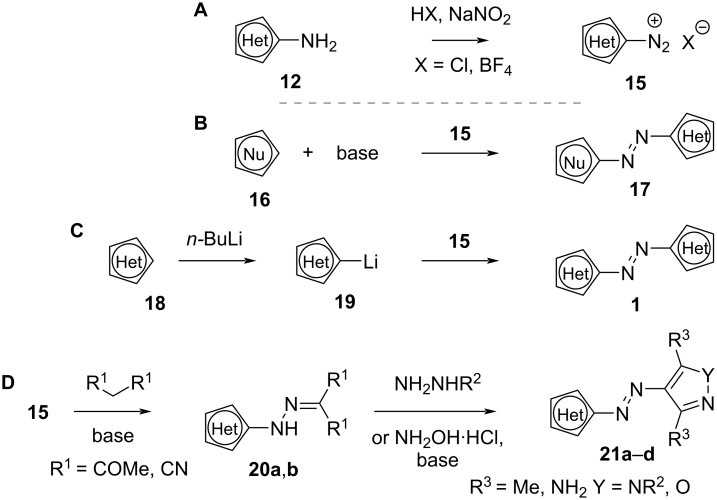
Synthesis of diazonium salt (A); different strategies of azo-coupling: with a nucleophilic ring (B), with a lithiated ring (C), and with a precursor (D).

Another strategy for both symmetric and asymmetric targets is the azo coupling of a diazonium salt **15** (Scheme 5A) with a nucleophile, which can be a (hetero)aromatic **16** [29] (B), a lithiated ring **19** [39] (C), or a precursor **20a**,**b** [29,32,40] (D). In case of more than one reactive position, the synthesis can be directed towards the desired target with the use of protecting groups [41].

**Scheme 6:**
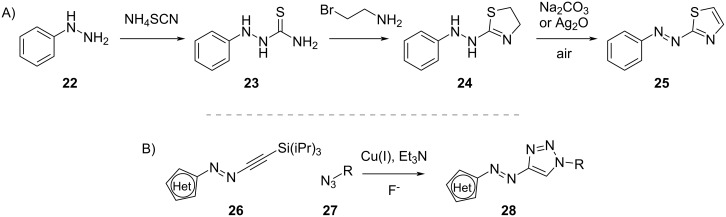
Synthesis of arylazothiazoles **25** (A) and heteroaryltriazoles **28** (B).

For the synthesis of azothiazoles **25**, the addition of phenylhydrazine (**22**) to ammonium thiocyanate followed by ring closure and oxidation was recently proposed (Scheme 6A) [42]. Heteroarylazo-1,2,3-triazoles **28** can be obtained by click chemistry (Scheme 6B) via one-pot deprotection of **26** and Cu(I)-catalysed reaction with an azide [43–44].

**Scheme 7:**
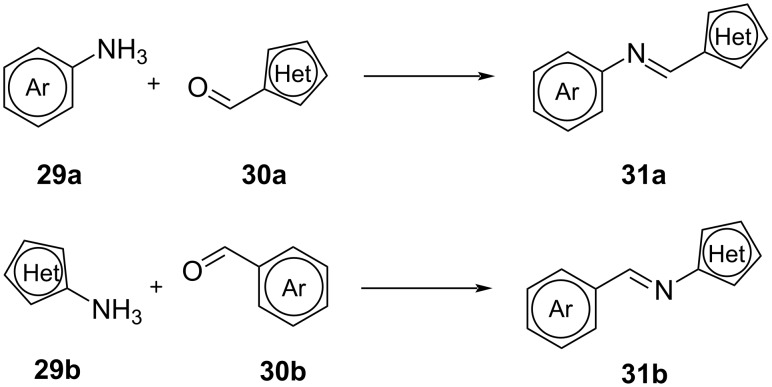
Synthesis of heteroarylimines **31a**,**b** [36–38].

Heteroarylimines **31a**,**b** can be easily obtained by condensation of a (hetero)aromatic aldehyde **30a**,**b** with a (hetero)aromatic amine **29a**,**b** [36–38] (Scheme 7). The choice of aldehyde and amine will determine the direction of the imine bond and the geometry of the *Z*-isomer.

#### Examples

**Figure 8:**
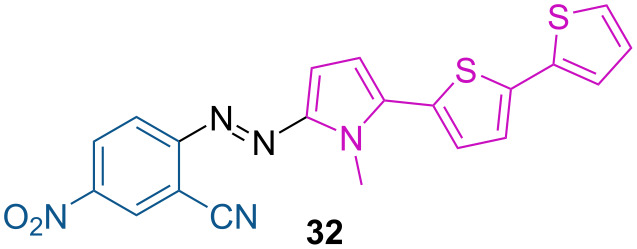
Push–pull non-ionic azo dye developed by Velasco and co-workers [45].

Non-ionic bithienylpyrrole push–pull azo dye **32** was successfully introduced in liquid-crystalline matrices with thermal relaxation in the µs order, making them among the fastest liquid crystalline optical oscillators (Figure 8) [45].

**Scheme 8:**
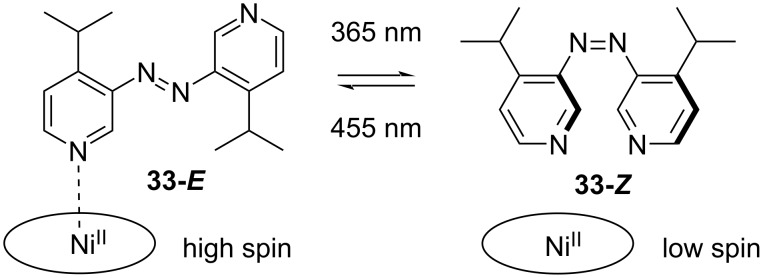
Azopyridine reported by Herges and co-workers [46].

The Herges group reported a photoinduced magnetic spin change of a Ni(II) porphyrine. The spin change was caused by the reversible coordination of azopyridine **33** to the Ni(II), which was only possible in the *E*-isomer (Scheme 8) [46].

**Scheme 9:**
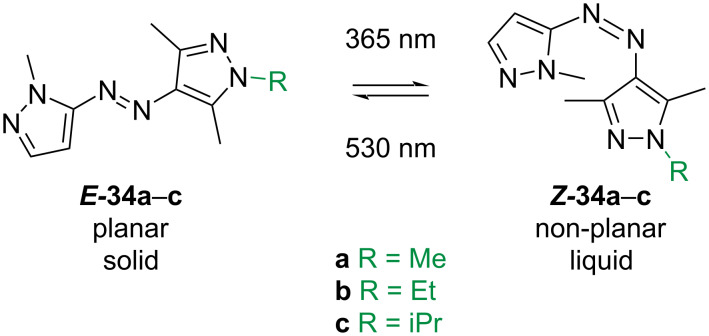
Photoinduced phase transitioning azobispyrazoles [47].

The Han group reported a series of compounds **34a**–**c** (Scheme 9) which melt upon irradiation with UV light in the solid phase. Upon irradiation with green light, the compounds isomerise back to the solid phase. The large depth of light penetration of these compounds (>1400 µm for both UV and vis light) and the high capacity for heat storage (>300 J/g) makes these compounds suitable for molecular solar thermal energy storage (MOST) [47].

### Diazocines

**Figure 9:**
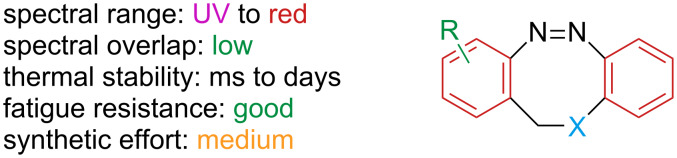
Diazocines.

Diazocines are bridged azobenzenes (Figure 9).

**Scheme 10:**
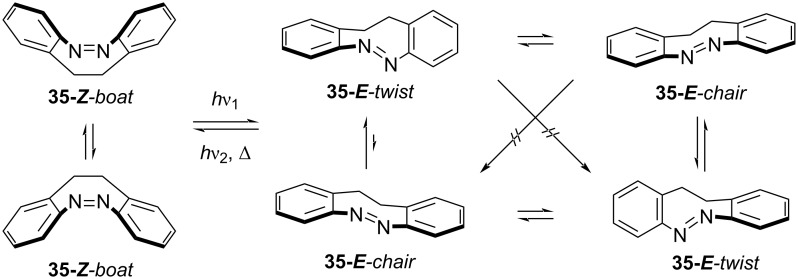
Isomers, conformers and enantiomers of diazocine.

The added strain renders the *E*-isomer metastable, favouring the more stable *Z*-isomer. In the planar *E-*azobenzene, the nπ* transition is symmetry-forbidden, which reflects in a low intensity of this band, while the *Z*-isomer is bent, giving rise to a higher intensity nπ* band [48]. The distorted nature of both *Z-* and *E*-isomers of diazocine makes the nπ* transition symmetry-allowed: as a consequence, the absorbance and the separation of the nπ* bands, which are used in the photoswitching process, are increased, leading to higher PSSs and higher excitation wavelengths, alongside with an excellent quantum yield in both directions. The bridge is also accountable for the much faster response to irradiation with respect to azobenzene [49]. The metastable *E*-diazocine exists in two conformers that can interconvert and have different absorption spectra (Scheme 10): *twist* (generally more stable) and *chair* (less stable). The spectrum of the chair conformer overlaps with the *Z*-isomer. Thus, to obtain better PSSs, the right conditions should be chosen to favour the twist conformer [50]. Each form exists in two enantiomers. The *E*-enantiomers cannot interconvert directly without first converting to the other conformer [51].

**Scheme 11:**
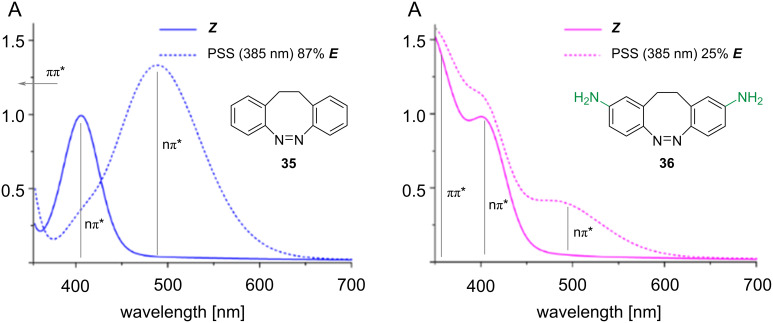
Partial overlap of the ππ* band with electron-donating substituents and effect on the PSS. Scheme 11 was adapted from [52] (© 2019 W. Moormann et al., published by Beilstein-Institut, distributed under the terms of the Creative Commons Attribution 4.0 International License, https://creativecommons.org/licenses/by/4.0).

Regarding the substitutions on the aromatic rings, it has been reported that electron-donating groups tend to red-shift the absorption of the ππ* absorption band, which overlaps with the nπ*, resulting in a decrease in the PSS (Scheme 11), while they are well tolerated when separated from the chromophore backbone [50,52–54]. Electron-withdrawing groups seem not to perturb much the absorption spectra [55]. There is one known report of diazocines with heteroaromatic rings: an electron-rich aromatic ring (thiophene) gives a similar effect to what is shown for electron-rich substituents with a very poor 385 nm PSS (18%), while an electron-poor aromatic as pyridine does not affect the absorption properties much [55].

**Figure 10:**
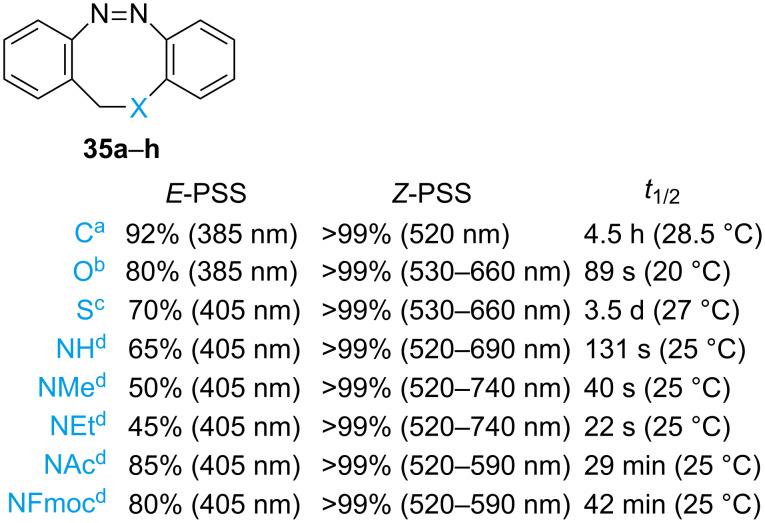
Main properties of diazocines with different bridges. ^a^Measured in *n*-hexane [56]. ^b^Measured in THF. ^c^Measured in acetonitrile [53]. ^d^Measured in acetone [51].

Several diazocines with heteroatoms in the bridge have been reported (Figure 10) [50–51,53]. Oxygen-containing heterodiazocine exhibits high PSS in both directions but has a short half-life, whereas the sulphur derivative has a significantly longer half-life but a slightly reduced *E*-PSS. This is likely due to the spectral overlap between the *E*-chair isomer and the *Z*-boat isomer [53]. Nitrogen-containing heterodiazocines were also reported. Alkyl substitution on N gave the highest bathochromic shifts. However, the *E*-PSS is generally low, again a probable consequence of the presence of the *E*-chair isomer [51]. All the compounds show excellent resistance to fatigue.

The behaviour of some (hetero)diazocines was also studied in water and solvent mixtures [50–51]. Interestingly, *N*-acetyl-diazocine possesses overall comparable properties and a longer thermal half-life, and it is also soluble in water. For *C*- and *S*-diazocine, the absorption band separation of the two isomers is lower in polar solvents, resulting in worse PSS. In particular, for the *S*-diazocine it was found that the *E*-chair conformer was bathochromically shifted with increasing polarity of the solvent until completely overlapping with the *Z*-boat conformer, making it impossible to switch it any further (42% *E* in H_2_O/MeCN 9:1). A diazonine was also synthesised, which exhibits a UV–vis spectrum of the *E*-isomer very similar to that of *E*-azobenzene and an unusually long *E–Z* thermal half-life [54].

#### Synthesis

**Scheme 12:**
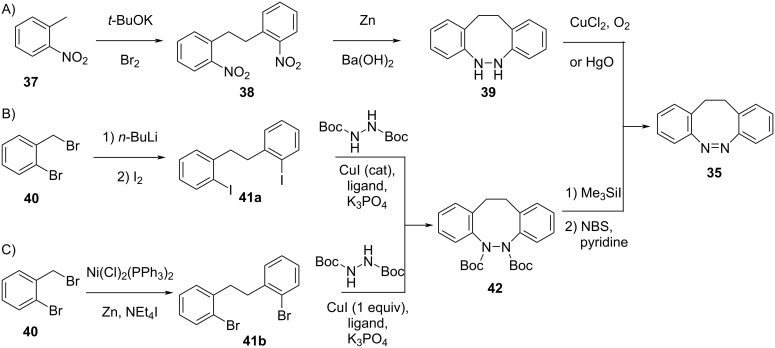
Synthesis of symmetric diazocines.

The synthesis of symmetrical diazocines is more demanding compared to classical azobenzenes since the two aromatic rings are not only connected via an azo-bond, but also have to be linked by an alkyl chain first. For that, more synthetic effort is required. It can be performed through oxidative dimerisation of *o*-nitrotoluene (**37**) followed by reduction with Zn/Ba(OH)_2_ and partial re-oxidation (Scheme 12A) [52]. They can also be obtained from *o*-halogenated benzyl bromides **40** by lithium–halogen exchange followed by nucleophilic substitution and a second lithium–halogen exchange with iodine (Scheme 12B) or by nickel-catalysed reductive cross-coupling of benzyl halides when substituents prone to reduction (CN, esters) are present (Scheme 12C). The Ullman–Goldberg coupling of **41b** with Boc-hydrazine followed by deprotection and oxidation then affords **35** [55].

**Scheme 13:**
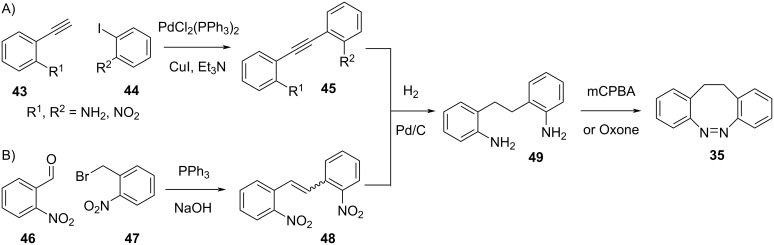
Synthesis of asymmetric diazocines.

Asymmetric diazocines can be synthesised by Sonogashira cross-coupling and subsequent reduction of **45** (if substrates **43** and **44** contain a NO_2_ group, this will also be reduced, Scheme 13A) [54], or by Wittig reaction and reduction of the isomeric mixture of alkenes **48** (Scheme 13B) [57]. Oxidation of **49** with oxone [58] or *m*-chloroperbenzoic acid [54] yields **35**. Wittig reaction can also be used to prepare precursors for the Ullman–Goldberg coupling (Scheme 12B and 12C); however, in the presence of halogens, an alternative reduction pathway of the alkene with tosylhydrazine and NaOAc must be used [55].

**Scheme 14:**
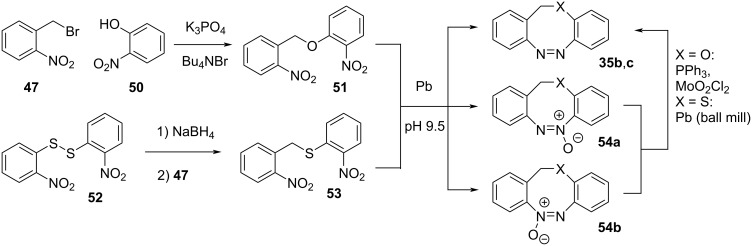
Synthesis of *O*- and *S*-heterodiazocines.

Hetereodiazocines **35b** and **35c** are synthesised by coupling of *o*-nitrobenzyl bromide (**47**) with **50** or with **52**, after its reduction with NaBH_4_. The intermediate products are then treated with lead in a buffered basic environment to get the final product in low yield accompanied by two by-products, **54a**,**b**. For the *O*-heterodiazocine, reduction with triphenylphosphine and a molybdenum catalyst allows partial conversion of the recovered by-product to the final product **35b**, while for the *S*-heterodiazocine lead was used for the reduction in neat conditions (Scheme 14) [53].

**Scheme 15:**
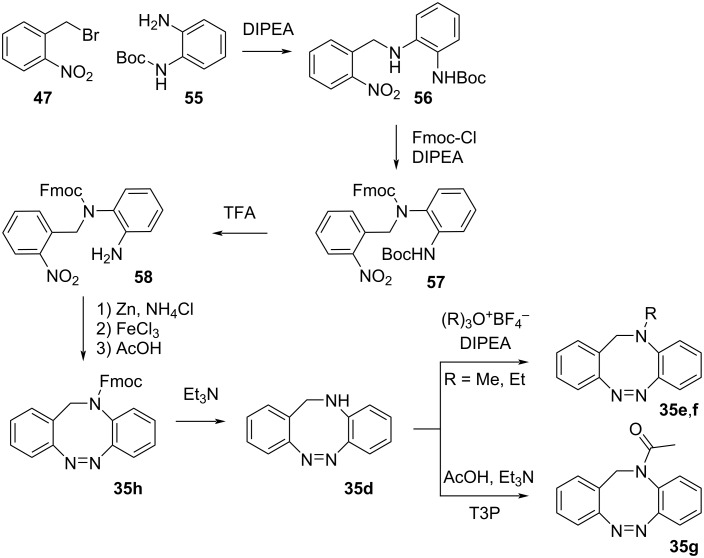
Synthesis of *N*-heterodiazocines.

*N*-Heterodiazocines (Scheme 15) can be synthesised by coupling of monoprotected diamine **55** with benzyl bromide **47**, followed by Fmoc protection of **56**, Boc deprotection of **57**, reduction of the NO_2_ group to NO and intramolecular Mills coupling to form **35h**. Removal of the Fmoc protecting group under basic conditions affords the unsubstituted product **35d** which, after *N*-alkylation or acylation affords the substituted products **35e**–**g** [51].

#### Examples

**Scheme 16:**
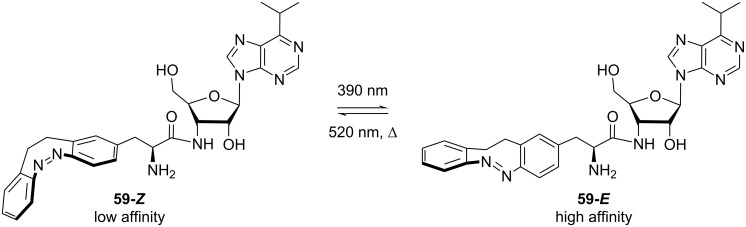
Puromycin diazocine photoswitch [60].

Because of their red-shifted absorption and inverse stability, diazocines are suitable substitutes for azobenzenes in biological environments. Light with longer wavelengths can penetrate biological media, such as tissue, more effectively, and is less harmful to living organisms than UV irradiation, for example. The inversion of *E*/*Z*-isomer stability compared to azobenzenes creates the opportunity to “invert” the mode of action in a biological context. Indeed, with azobenzene-based photopharmacophores, it is mostly the case that the least bulky *E*-isomer is a better fit in the protein-binding site than the bent *Z*-isomer. However, it would be more desirable to have an inactive molecule in its most stable state which can be activated by irradiation [51,59]. Hernando and co-workers were able to design photoswitchable neurotransmitters of ionotropic kainate receptors by replacing the azobenzene moiety of the already established partial agonist GluAzo with a diazocine unit. While both photochromic ligands show biological activity in the *E* form, the *E*-isomer is the thermodynamically more stable one for the azobenzene analog, whereas for the diazocine-modified neurotransmitter it is the *Z* form. Hence, GluAzo would permanently show biological activity in the dark (*E*-isomer), whereas the diazocine analog (*Z*-isomer) would be inert and only gets activated upon irradiation [58]. The Trauner group synthesised a puromycin diazocine **59** that shows higher affinity to the ribosome pocket in its (metastable) *trans*-isomer, inhibiting RNA translation (Scheme 16). In contrast, the azo-puromicin did not show significant change in activity between the two isomers [60].

### Indigoids

**Figure 11:**
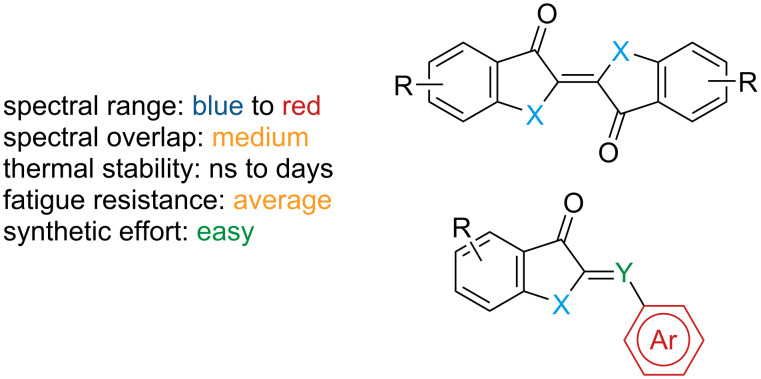
Indigoids.

Indigoid photoswitches are characterised by switching in both directions with visible light (Figure 11).

**Figure 12:**
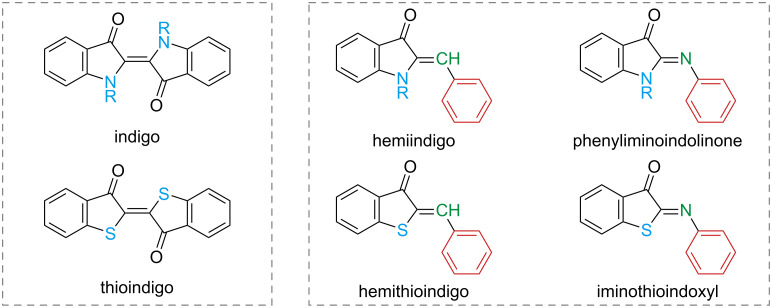
The main representatives of the indigoid photoswitch class.

The main representatives are shown in Figure 12. The relative stability of the isomers is generally *E* for indigo and thioindigo and *Z* for the other indigoids, but there are a few exceptions that depend on the substitution pattern [61–64]. Due to the very different properties, each subclass should be treated separately.

**Scheme 17:**
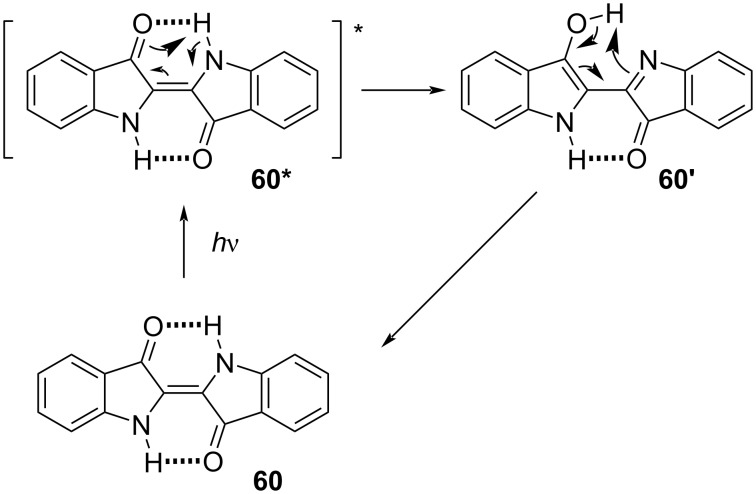
Deactivation process that prevents *Z*-isomerisation of indigo.

**Figure 13:**
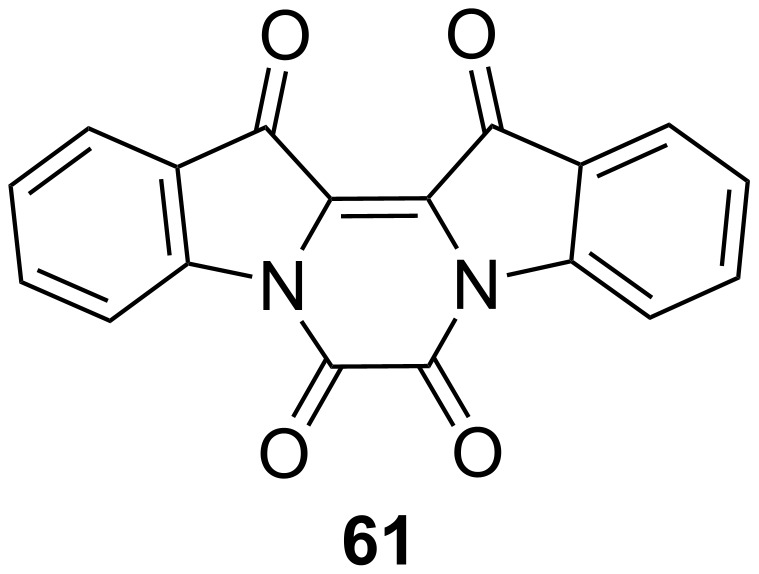
Stable *Z*-indigo derivative synthesised by Wyman and Zenhäusern [67].

Indigo and thioindigo possess an intrinsic push–pull character, giving them extremely red-shifted spectra without the need for additional substituents [65]. The *E*-isomer is planar and conjugated, while the *Z*-isomer is not. The rupture of conjugation causes a hypsochromic shift, which characterises this subclass with negative photochromism. Unsubstituted indigo (**60**) does not undergo photochemical isomerisation, due to much faster relaxation through excited-state proton transfer 60* followed by tautomerisation **60’** (Scheme 17). The first proof of the existence of *Z-*indigo was provided by Wyman and Zenhäusern, who isolated derivative **61**, which is sterically constrained to the *Z* form, and compared the absorption spectrum to the spectra of already reported *Z*-indigoids (Figure 13) [66].

**Figure 14:**
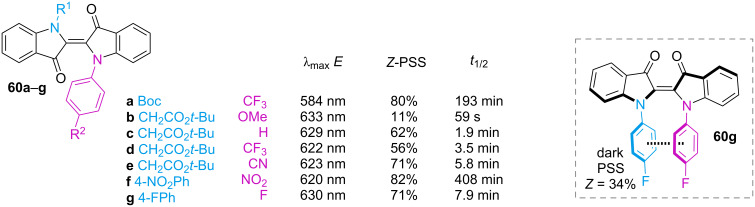
Selected examples of indigos with aliphatic and aromatic substituents [68]. Dashed box: proposed π–π interaction stabilising the diaryl-substituted derivatives.

The Hecht group rationalised the role of *N*-substitution on the photophysical properties of indigos [67]. In general, electron-donating substituents red-shift the absorption and decrease the thermal half-life, and vice versa for the electron-withdrawing ones. Phenyl rings with electron-withdrawing *p*-substituents allow a better fine-tuning of the spectral separation between the two isomers (thus, giving better PSSs) and improved the half-lives without interfering too much with the absorption of the *E*-indigo (Figure 14). Diarylated indigos like **60g** showed the presence of the *Z*-isomer in the dark, possibly due to the stabilisation energy given by the π–π interaction between the aryl rings (Figure 14, box) and repulsion between the aryl and the carbonyl group in the *E*-isomer.

**Scheme 18:**
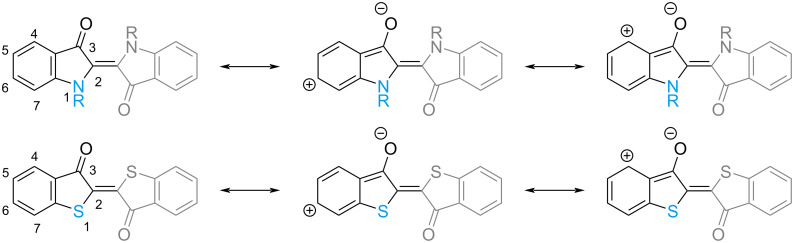
Resonance structures of indigo and thioindigo involving the phenyl ring.

Some studies on *N*-acylindigo derivatives reported, besides the expected spectral blue-shift, also a decrease in the fatigue resistance due to photochemical rearrangements [68–69]. Dube and co-workers reported a series of mono-*N*-substituted indigos which were also able to isomerise, despite the presence of one hydrogen atom, capable of proton transfer. Only rather electron-neutral substituents show photoswitching to some extent, and the thermal half-lives are, in general, very short. Interestingly, low amounts of water led to a strong decrease in the thermal half-life, suggesting water is also involved in the *Z–E* thermal isomerisation process [70]. Bridged indigos have also been reported for which the *Z*-isomers are unstable. By bridging the two nitrogen atoms, these compounds show planar chirality and can be racemised upon irradiation. For further details about bridged indigos, we refer the reader to the work of Tsubaki and co-workers [71]. Although less pronounced, substituents in the phenyl ring also change the absorption properties of indigos. Considering only the resonance structures that involve the phenyl ring (in Scheme 18), one can conclude that electron-withdrawing groups in positions 4 and 6 and electron-donating groups in positions 5 and 7 decrease the energy of the excited state, resulting in a bathochromic shift. Conversely, electron-donating groups in positions 4 and 6 and electron-withdrawing groups in positions 5 and 7 destabilise the excited state and give hypsochromic shifts [72–73]. Positions 4 and 7 also have some steric effects [65].

**Scheme 19:**
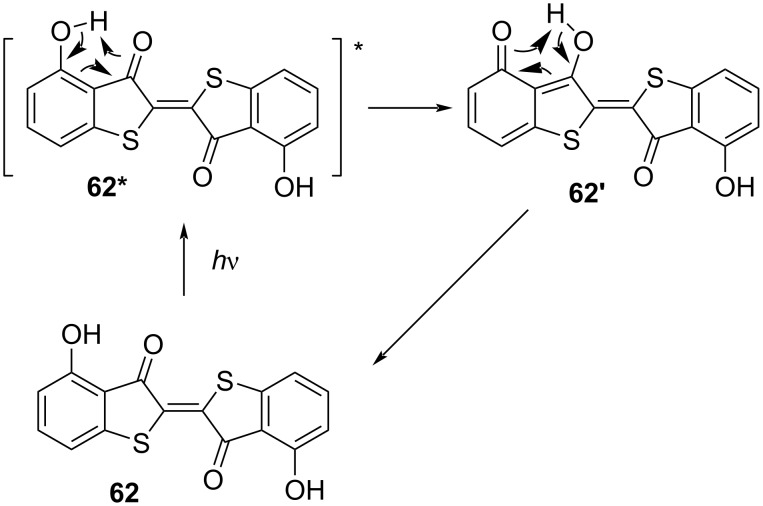
Possible deactivation mechanism for 4,4'-dihydroxythioindigo [76].

The impossibility of proton transfer in thioindigo makes it readily switchable without the need for any substituents on the sulphur atom. The same effects of ring substitution as indigo are observed (Scheme 18) [73–74]. A complete quench of thioindigo isomerisation was obtained by the addition of OH groups in the 4-position [75]. Upon excitation of **62**, it is believed a similar deactivation mechanism to the unsubstituted indigo is operative (Scheme 19). By exchanging both OH groups with methoxy, the switching properties are fully restored.

**Scheme 20:**
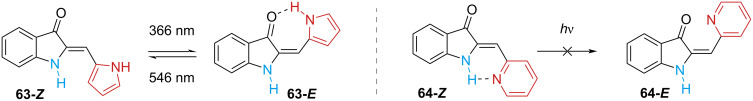
Effect of different heteroaryl rings on the stability and the photophysical properties of hemiindigos [61–62].

Preliminary studies on hemiindigo reported dimerisation as a side reaction of irradiation with UV light during their synthesis. This was particularly evident in substrates with electron-donating substituents on the stilbene fragment [76]. Recently, hemiindigos with electron-donating substituents were rediscovered by the Dube group, which reported all-vis photoswitches with very high PSS in both ways and good to excellent thermal stabilities. Lower absorption coefficients of the *Z*-isomers in the derivatives with bulky *N*-substitution suggest that the conformation is twisted, with reduced conjugation. Steric hindrance at the indoxyl nitrogen also provides higher thermal stability of the *E*-isomer, with mixtures of *E* and *Z* found at thermal equilibrium [77]. Heteroaromatic substitution in hemiindigo generates interesting results: in the case of derivative **63** containing pyrrole, capable of hydrogen bonding with the carbonyl oxygen (Scheme 20, left) [61–62], the *E* form is obtained almost quantitatively upon irradiation. In the case of pyridine-containing derivative **64** (Scheme 20, right) no photoswitching is observed as a consequence of a deactivation pathway similar to that of indigo [62].

**Figure 15:**
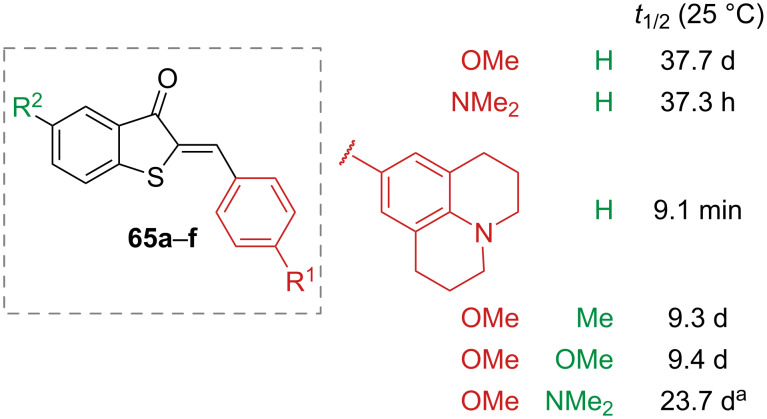
Thermal half-lives of red-shifted hemithioindigos in toluene [79]. ^a^Measured in toluene-*d*_8_.

The effect of substituents in hemithioindigo has been extensively studied. The presence of electron-donating groups in the *o*- and *p*-position of the phenyl ring generates hemithioindigos with very fast response to irradiation, but very strong electron donors were found to slow down the isomerisation, which was also complemented by a strong red-shift of the absorption spectrum [78]. Moreover, the thermal stability is also affected. To obtain red-shifted photoswitches, which are also thermally stable, electron-donating groups can be added to the thioindigo fragment in *p*-position to the sulphur atom (Figure 15) [79].

**Scheme 21:**
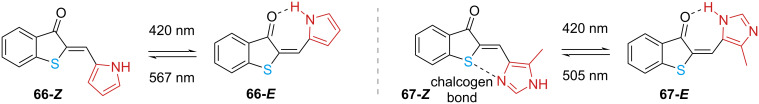
Structures of pyrrole [81] and imidazole hemithioindigo [64].

Substituents in the *o*-position generate twisted hemithioindigos, which do not switch in polar solvents, due to an alternative deexcitation pathway [80]. Heteroaryl substituents are also reported: a pronounced red-shift of the *E*-isomer was observed for the pyrrole derivative **66** [81], showing that the hydrogen bond between pyrrole and carbonyl also plays a role (Scheme 21, left). The band separation also provides almost quantitative PSS in both directions. The imidazole derivative **67** (Scheme 21, right) can interchange between tautomers to make a chalcogen bond with sulphur in the *Z*-isomer and a hydrogen bond in the *E*-isomer [64]. Extension of the conjugation with additional electron-rich arenes enables isomerisation with wavelengths up to >700 nm, with high PSS and high quantum yield. Electron-poor heterocycles generally possess strongly overlapped spectra with low PSS and low resistance to fatigue [64,82]. The hydrogen bond was also recently used to generate photobases [83].

**Figure 16:**
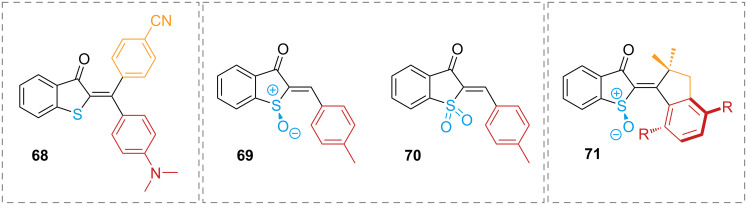
Examples of fully substituted double bond hemithioindigo (left), oxidised hemithioindigos (centre), and a sulphoxide hemithioindigo as a molecular motor (right).

Hemithioindigos with fully substituted double bonds have also been reported: more rigid stiff stilbene moieties have been explored [84–85], as well as alkyl and aryl groups [86–87]. The diaryl hemithioindigos **68** offer the most interesting properties, namely improved thermal half-lives, red-shifted absorption spectra, and higher molar extinction coefficients, with a combination of electron-withdrawing substituents on one side and electron-donating substituents on the other side giving the best performance (Figure 16, left). These peculiar compounds also showed increased stability and red-shift with increasing solvent polarity [87]. Oxidation of the sulphur atom to sulphoxide **69** introduces a sulphur-based stereocentre, which, combined with additional steric hindrance to the stilbene moiety and on the double bond, induces a helical twisting around the double bond in **71**, thus generating a molecular motor, capable of unidirectional movement upon photoswitching (Figure 16, right) [84–85]. Sulphone hemithioindigos **70** have also been explored. Unlike sulphoxides, sulphones are not chiral. Both sulphone and sulphoxide are more electron-deficient than hemithioindigo, which induces a blue-shift of the absorption spectrum (Figure 16, centre) [88].

**Scheme 22:**
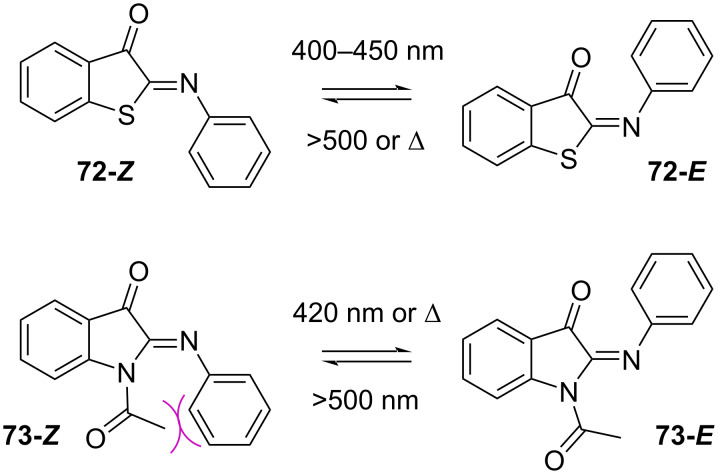
Structure of iminothioindoxyl **72** (top) and acylated phenyliminoindolinone photoswitch **73** (bottom). The steric hindrance in **73-*Z*** makes it the least stable isomer and is responsible for the negative photochromism.

**Scheme 23:**
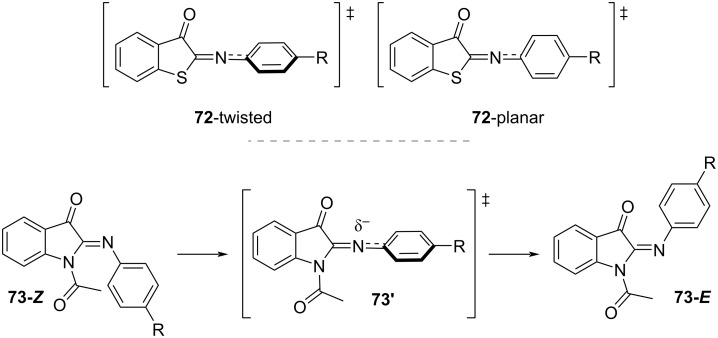
(top) Transition states of iminothioindoxyl **72**. The planar transition state is associated with a longer thermal half-life [90]. (bottom) Transition state for thermal back-isomerisation of phenyliminoindolinone **73**. The partial negative charge in **73’** is stabilised by resonance with electron-withdrawing groups –R [63].

The photophysical properties of iminothioindoxyl **72** (Scheme 22, top), although already known as dye [89] have only been studied very recently. Exchanging the C=C bond for a C=N bond gives a new subclass of extremely short-lived T-type photoswitches with visible light activation, thermal half-lives in the µs range, and very large spectral separation. The study of iminothioindoxyl with different substituents on the phenyl 4-position shows that electron-donating groups generate bathochromic shifts as well as increase the absorption coefficient. This is explained by the increased electron density of the phenyl ring, which tends to planarise and to extend the conjugation to the rest of the molecule. The transition state for the thermal back-isomerisation of **72** is planar in case of electron-donating groups and twisted for electron-withdrawing groups; the latter isomerise faster than the former (Scheme 23, top). The planar transition state is also favoured in polar and protic solvents, causing a slower thermal isomerisation. The photoswitch is suitable for aqueous buffers and biological environments and is resistant to glutathione oxidase [90]. Phenyliminoindolinone **73** was designed to improve the features of iminothioindoxyl [63]. Substitution at the indole nitrogen of phenyliminoindolinone with an acetyl group destabilises the *Z*-isomer due to the steric hindrance of the substituent, giving negative photochromism (Scheme 22, bottom). Bulkier substituents at the indole nitrogen do not influence much the photophysical properties, while electron-withdrawing groups in the phenyl 4-position give hypsochromic shifts and shorten the thermal half-life by stabilising the transition state **73’** (Scheme 23, bottom) [63].

#### Synthesis

**Scheme 24:**
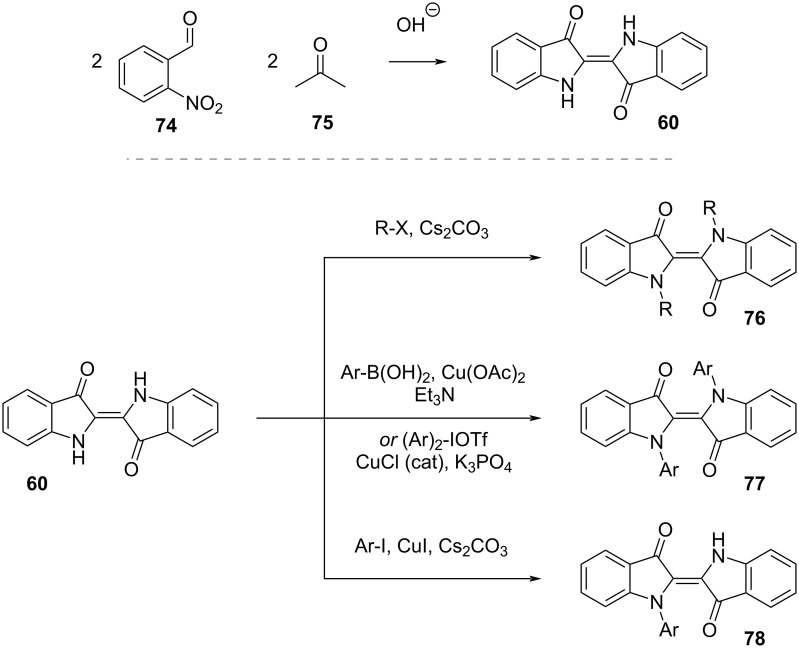
Baeyer–Drewsen synthesis of indigo (top) and *N*-functionalisation strategies (bottom).

The classic synthesis of indigo is achieved via Baeyer–Drewsen synthesis reacting 2-nitrobenzaldehyde (**74**) and acetone in basic medium (Scheme 24, top) [91]. Since indigo is a well-known commercial product, we will not focus on other synthetic pathways, which can be found in a review by Hecht and co-workers [65], and instead discuss the *N*-functionalisation (Scheme 24, bottom). *N*-Alkylation in **76** can be achieved with alkyl halides and base. There are different methods for *N*-arylation depending on the aryl type: electron-rich and electron-neutral substituents are introduced via Chan–Lam coupling with an arylboronic acid, electron-poor aromatics via Cu(I)-catalysed cross-coupling with aryliodonium salts, and monosubstitution is achieved via Ullman–Goldberg coupling with aryl iodides. The monosubstituted product **78** can be further functionalised with any of the previous methods [67].

**Scheme 25:**
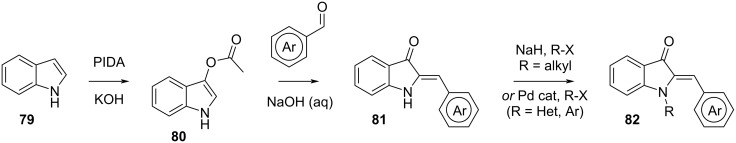
Synthesis of hemiindigo.

Hemiindigos **81** and **82** are synthesised by generation of **80** via reaction with (diacetoxyiodo)benzene (PIDA) in the presence of base, followed by reaction with the corresponding aldehyde and, if required, *N*-functionalisation via nucleophilic substitution (for aliphatic substituents) or palladium-catalysed cross-coupling (for aromatic substituents) (Scheme 25) [77].

**Scheme 26:**
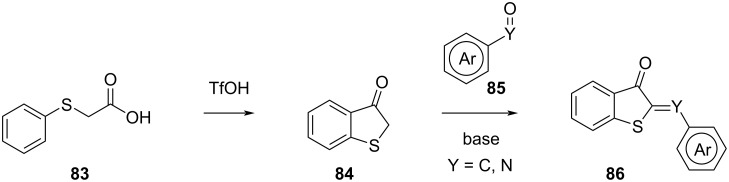
Synthesis of hemithioindigo and iminothioindoxyl.

Hemithioindigo can be synthesised by treating phenylthioacetic acid (**83**) with triflic acid. Then, the product is condensed with a (hetero)aromatic aldehyde in the presence of a base to yield **86** (Scheme 26) [81]. Iminothioindoxyl can be synthesised in the same way, using nitrosoarenes instead of aldehydes [90]. For several other synthetic methods, we refer to the excellent reviews by Konieczny and Konieczny [92] and Wiedbrauk and Dube [93].

**Scheme 27:**
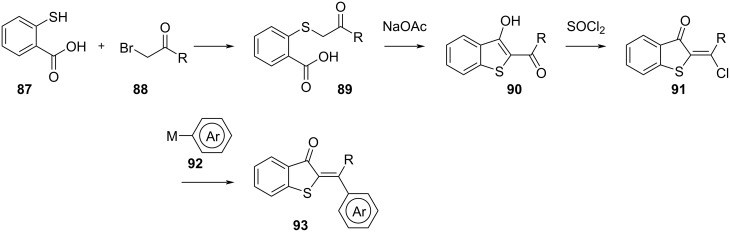
Synthesis of double-bond-substituted hemithioindigos.

Double-bond-substituted hemithioindigos **93** are synthesised by functionalising **87** with an aliphatic or aromatic **88** and subsequent intramolecular aldol reaction to **90**. Treatment with SOCl_2_ and with the aromatic nucleophile or a cross-coupling partner of choice affords **93** (Scheme 27) [86–87].

**Scheme 28:**
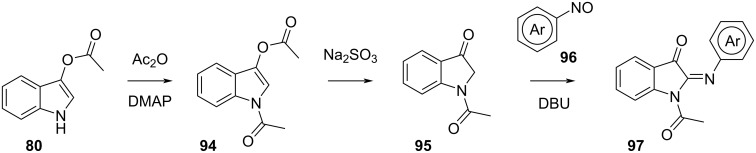
Synthesis of phenyliminoindolinone.

Phenyliminoindolinone is synthesised by *N*-acetylation of **80** with acetic anhydride catalysed by DMAP, then **94** is treated with sodium sulphite to give **95.** Base-assisted coupling with a nitrosoarene finally yields *N*-acetylphenyliminoindolinone **97** (Scheme 28) [63].

As each subclass shown in this chapter requires individual synthetic pathways, it is difficult to simply classify the synthetic accessibility of indigoids in general. For example, while it is straightforward to get *N*-functionalised indigos, it requires more synthetic effort for double-bond-substituted hemithioindigos. Hence, depending on the desired indigoid photoswitch, synthesis may show varying levels of difficulty. Nevertheless, if compared to diarylethenes or fulgides, indigoids are typically easier to obtain.

#### Examples

**Scheme 29:**
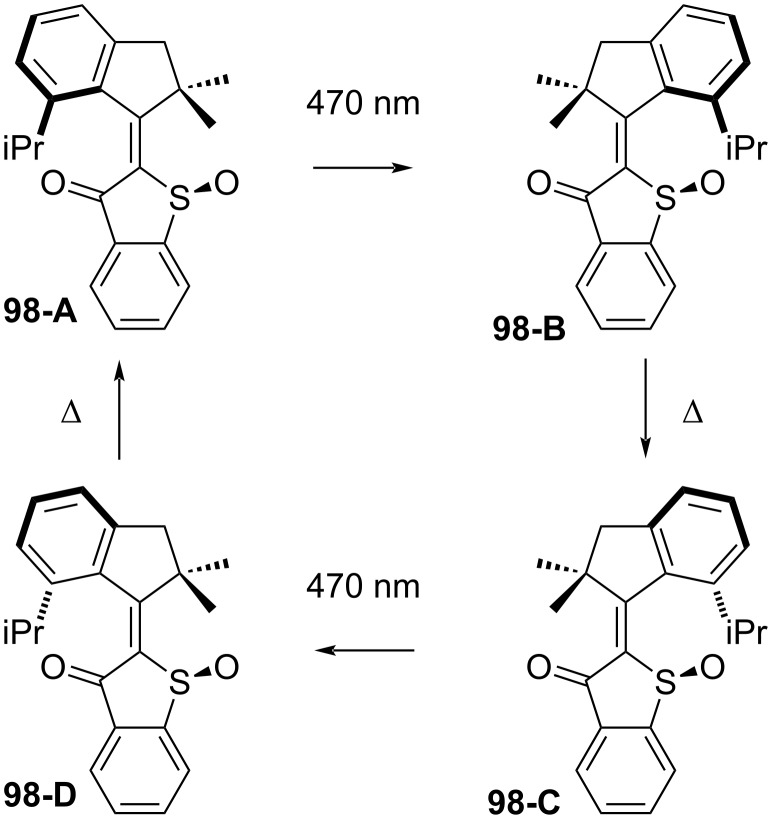
Hemithioindigo molecular motor [85].

The chiral hemithioindigo sulphoxide **98** has been demonstrated to have unidirectional rotation upon photoisomerisation, making it an all-visible example of a molecular motor (Scheme 29) [85]. Irradiation at 470 nm of **98-A** leads to the thermally unstable **98-B,** which immediately converts to the less sterically hindered **98-C**. Further irradiation leads to the thermally unstable **98-D**, which again converts quickly to the starting point **98-A**. The unstable isomers were observed at low temperatures. Besides that, hemithioindigos have been used to operate as structural switches in peptides. Upon irradiation, the photoisomerisation of the hemithioindigo moiety is triggered, leading to an overall structural change of the peptide chain [94].

### Arylhydrazones

**Figure 17:**
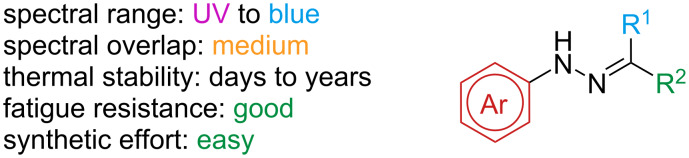
Arylhydrazones.

Arylhydrazones are a class of extremely versatile photoswitches, with good fatigue resistance and thermal half-lives that span from a few hours to years (Figure 17).

**Scheme 30:**
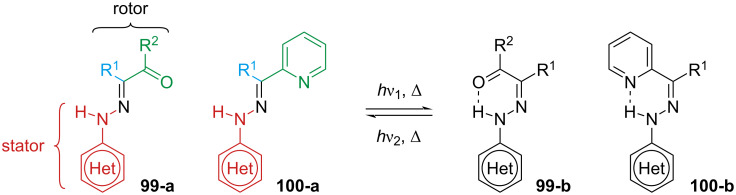
Switching of arylhydrazones. Note: The definitions of stator and rotor are arbitrary.

The common feature of arylhydrazones is the presence of a H-bond acceptor in the rotor, which stabilises one of the two isomers via the formation of a hydrogen bond (Scheme 30) [95]. This peculiarity makes this photoswitch class extremely susceptible to solvent polarity [96–98], pH [98–101], and metal coordination [102–104].

**Scheme 31:**
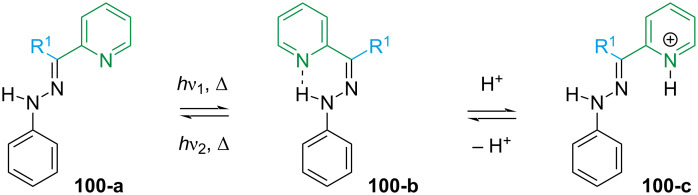
Photo- and acidochromism of pyridine-based phenylhydrazones.

With such variability, predicting which is the most stable isomer is not always intuitive, and several early studies focused on this aspect [96,105–106]. For small substituents (H, Me, CN) in aprotic solvents and in the absence of other external factors, the equilibrium is usually shifted towards the least hindered configuration, regardless of the presence of H-bond acceptors [102,104,107]. The presence and the nature of the H-bond can be identified by ^1^H NMR spectroscopy since the N–H proton has a characteristic chemical shift depending on the H-bond acceptor [101]. Arylhydrazones based on pyridine have been intensively studied since the H-bond between hydrazone and pyridine can be broken by protonation with an acid and even by metal coordination, rendering these compounds photo- and acidochromic (Scheme 31) [102]. The equilibrium can also be modulated by introducing hydrogen-bond acceptors in R^1^ [107] or by introducing substituents to the stator [95].

**Scheme 32:**
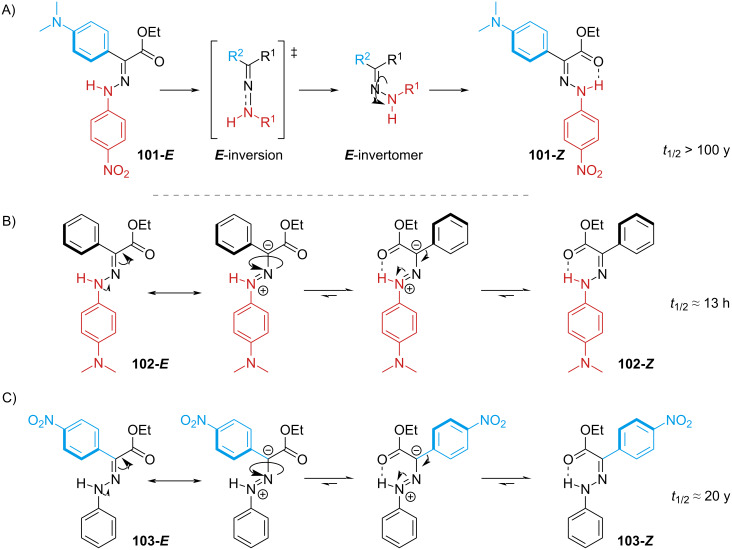
A) *E–Z* thermal inversion of a thermally stable push–pull hydrazone [109]. B) Rotation mechanism favoured by electron donation from the stator. C) Rotation mechanism favoured by electron withdrawal from the rotor. The effect of the rotor is milder because of the nonplanar structure.

The photoswitching of arylhydrazones was first reported in 1976 by the group of Courtot [96,105], and the structure–property relationships were thoroughly studied by the Aprahamian group [108]. Electron-donating groups generally give a red-shifted spectrum, while electron-withdrawing groups (with the exception of NO_2_) do not change the absorption significantly with respect to unsubstituted derivatives. It is important to note that, unlike the azo-bond, the hydrazone bond is not symmetric, which means that not only the type of substituent but also the position will strongly influence the physical properties. Combining *p*-NO_2_ on the stator with a *p*-NMe_2_ group at the rotor affords the red-shifted hydrazone **101** with almost a quantitative PSS in both directions, while not affecting the thermal stability much (Scheme 32A). Conversely, the presence of an electron-donating group (NMe_2_ or OMe) in the stator shortens the thermal half-life due to a change in mechanism, from inversion to rotation around the C=N bond (Scheme 32B). Due to the direct conjugation of NMe_2_ with the hydrazone core, both isomers of **102** are red-shifted, resulting in strong spectral overlap which gives only 27% of the *E* form upon photoswitching and no suitable wavelength was found for *E–Z* photoisomerisation. Compound **103** with an electron-withdrawing group on the rotor also has a shorter half-life. However, the effect is mitigated by the fact that the rotor is not perfectly planar with respect to the hydrazone, which leads to a less effective conjugation (Scheme 32C). The lower degree of conjugation also reflects in a less pronounced red-shift and spectral overlap compared to **102**, and a better PSS (*Z–E* 92%, *E–Z* 52%).

**Scheme 33:**
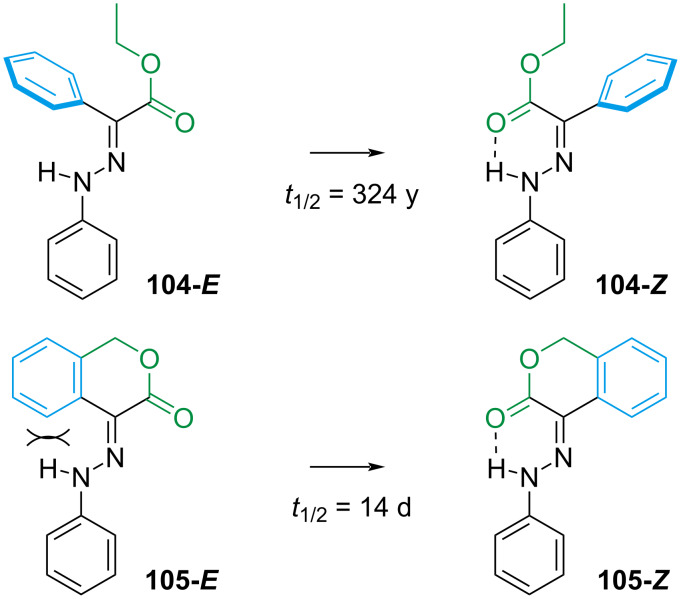
Effect of planarisation on the half-life.

This hypothesis was further supported by forcing the planarity of the rotor phenyl by ring strain (Scheme 33) [110]. The direct consequence of the planar **104** is the enhanced conjugation, which gives a red-shift in the absorption and favours the rotation mechanism (as seen in Scheme 32). The forced steric interaction of the aryl ring with the hydrazone moiety destabilises **105-*E*** even further, decreasing the thermal stability from hundreds of years to a few days.

**Scheme 34:**
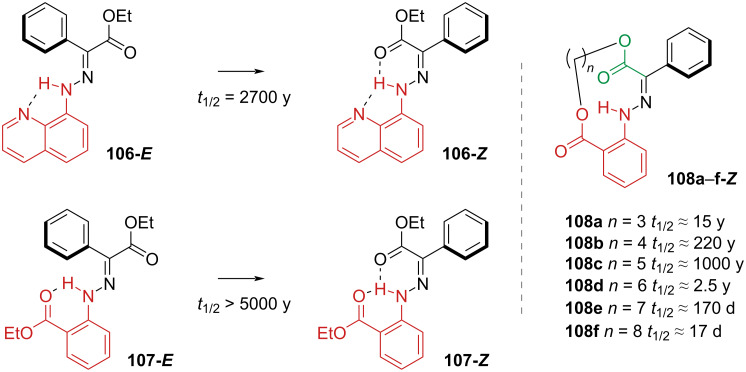
The longest thermally stable hydrazone switches reported so far (left). Modulation of thermal half-life through ring size (right).

The addition of a further hydrogen-bond acceptor on the stator led to the discovery of hydrazones with a thermal stability of thousands of years (Scheme 34, left) [111]. The extra H-bond in **106-*E*** and **107-*E*** raises the energy of the linear transition state *E*-inversion (Scheme 32A). The addition of ring strain between the stator and the rotor (Scheme 34, right) was found to affect the thermal half-life depending on the ring size [109]. Too small (*n* = 3) **108a** and too large rings (*n* > 5) **108d**–**f** force the *E*-isomer in a less stable conformation and weaken the hydrogen bonds between N–H and the ester moieties of the rotor and the stator.

**Figure 18:**
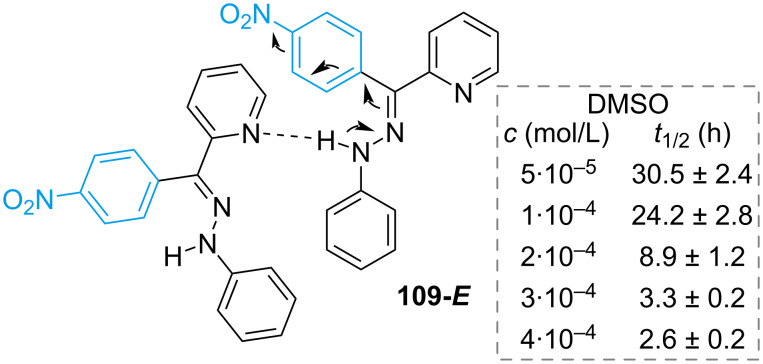
Dependency of *t*_1/2_ on concentration and hypothesised aggregation-induced isomerisation.

The Cigáň group focused on the synthesis of benzoylpyridine hydrazones with different substitutions in the rotor benzene [97]. The *p*-NMe_2_ group causes red-shift of both absorption maxima of the *E*- and *Z*-isomer, however, it leads to a poor *Z–E* PSS and poor resistance to fatigue. This is most likely due to internal relaxation processes prevailing over photoswitching. One interesting finding was the strong dependency of the thermal half-life on concentration in DMSO. The authors hypothesised the aggregation of **109-*E*** (Figure 18) that leads to a weakening of the C=N bond and consequent rotation mechanism (similar to **103** in Scheme 32C). This hypothesis was corroborated by the same trend in the presence of the base Et_3_N.

**Figure 19:**
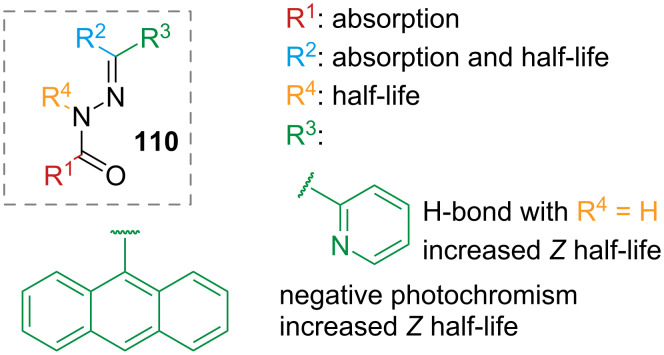
Structure–property relationship of acylhydrazones.

Acylhydrazones **110** are also worth mentioning in this section for their structural and synthetic similarity to arylhydrazones. Their structure–property relationships were thoroughly studied by the Hecht group, focusing in particular on the maximum absorption of the *E*-isomer, on the band separation between *E* and *Z*, and on the thermal half-life [112]. Electron-rich substituents in R^1^ (Figure 19) result in slight bathochromic shifts of the spectra, while substitution of hydrogen for methyl in R^2^ has a slight hypsochromic effect on the absorption maximum of the *E*-isomer, and the thermal half-life is generally shorter. Methylation of N (R^4^) causes the destabilisation of the *E*-isomer and increased thermal stability of the *Z*-isomer. The most interesting effects were found by variation of R^3^: increasing the conjugation with large aromatic moieties shifts the absorption maximum of the *E*-isomer to lower energies with respect to the *Z*-isomer, giving negative photochromism, and also increases the thermal half-life. Substitution with heteroaromatics affords changes in the absorption maxima and in the band separation, but no clear trend was observed. The introduction of an H-bond acceptor (2-pyridine) increases the lifetime of the *Z*-isomer when R^4^ is a hydrogen atom.

#### Synthesis

**Scheme 35:**
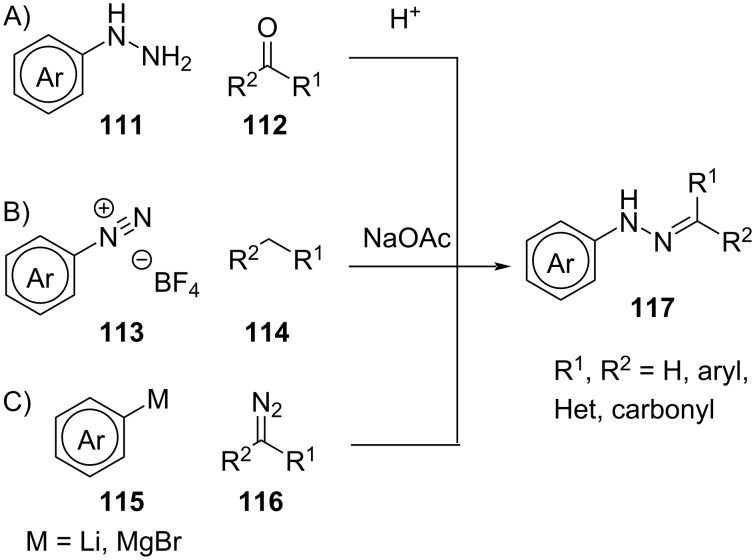
Synthesis of arylhydrazones.

In general, aryl- and acylhydrazones are accessible with comparatively low synthetic effort, especially compared to diarylethenes and fulgides. The synthesis of arylhydrazones is usually performed through condensation between a carbonyl **112** and a hydrazine **111** (Scheme 35A) [109]. Alternatively, one can perform an azo-coupling between a diazonium salt **113** and a nucleophile **114** (Scheme 35B) [113] or a Grignard/aryllithium reagent **115** and a diazo compound **116** (Scheme 35C) [108].

**Scheme 36:**
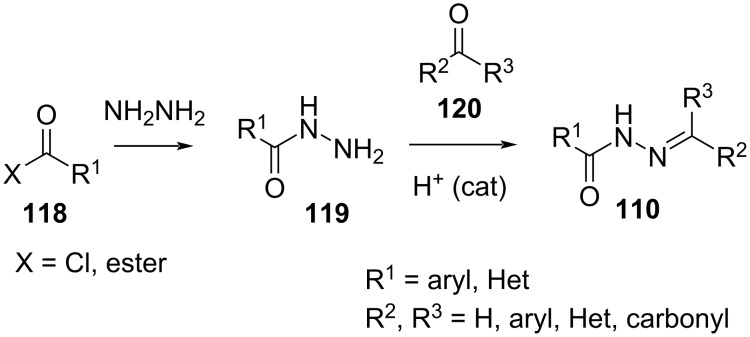
Synthesis of acylhydrazones.

Acylhydrazones **110** can be synthesised by reaction of hydrazine with **118** and subsequent acid-catalysed condensation of **119** with carbonyl **120** (Scheme 36) [112].

#### Examples

**Scheme 37:**
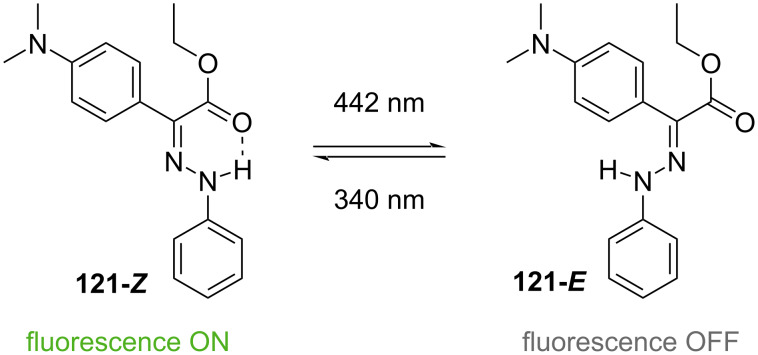
Photoswitchable fluorophore by Aprahamian et al. [115].

The Aprahamian group reported hydrazone **121**, which is emissive in its *Z*-isomer. The fluorescence can be switched on and off by irradiation with 442 nm and 340 nm, respectively (Scheme 37) [114]. The photophysical and emissive properties were also maintained in bovine serum, in the solid state, and in the presence of glutathione, opening the possibility for applications in a biological environment.

**Scheme 38:**
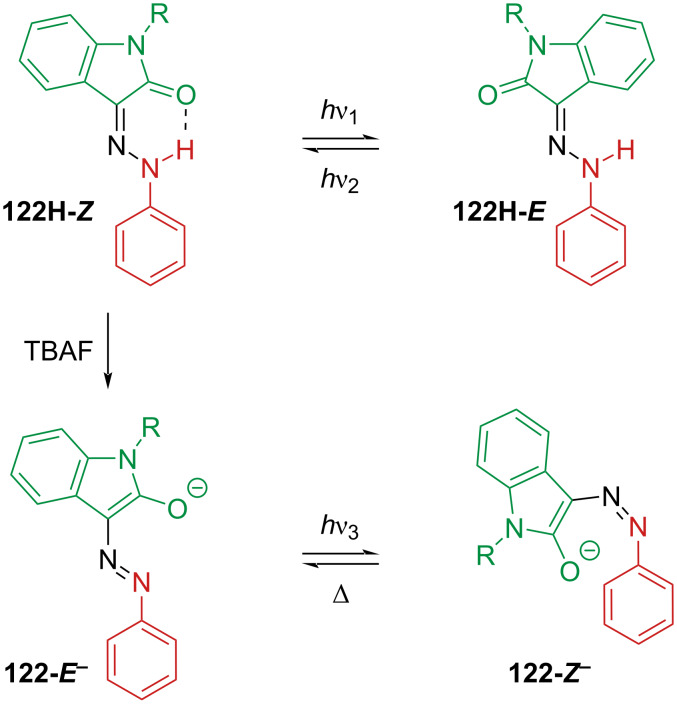
The four-state photoswitch synthesised by the Cigáň group [116].

Some interesting isatin-based arylhydrazones were reported [115–117]. Compound **122H** is a P-type hydrazone photoswitch. The addition of tetrabutylammonium fluoride (TBAF) deprotonates the hydrazone, affording the T-type azo-switch **122-*E^−^*** (Scheme 38).

### Diarylethenes

**Figure 20:**
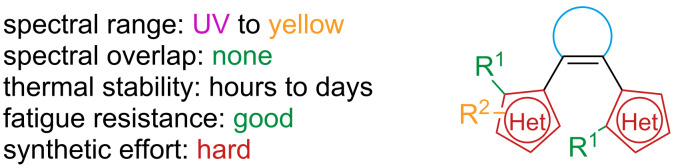
Diarylethenes.

Diarylethenes are a fascinating class of robust photoswitches. Their main features include thermal stability, a high quantum yield of cyclisation, excellent resistance to fatigue, and a picosecond-range response to irradiation [118] (Figure 20).

**Scheme 39:**
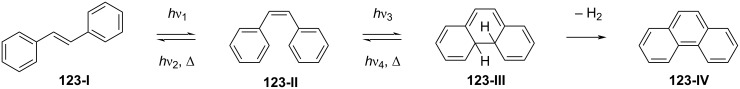
Isomerisation and oxidation pathway of *E*-stilbene to phenanthrene.

The primary example of a diarylethene is stilbene: it was discovered that upon irradiation, stilbene would not only switch between the *E*- (**I**) and *Z*-configuration (**II**) but also, in its *Z*-configuration, it would undergo a 6π-electrocyclisation (**III**) (see Scheme 39) [119]. However, this form is not stable, and it easily undergoes irreversible oxidation under air to give phenanthrene (**IV**).

**Scheme 40:**
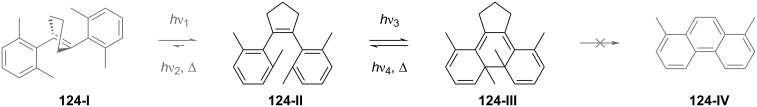
Strategies adapted to avoid *E–Z* isomerisation and oxidation.

This challenge was addressed by substituting the hydrogen atoms with methyl groups in **124-II**. Moreover, the *E*–*Z* isomerisation could be avoided by adding a ring to the central double bond, thus destabilizing the *E*-isomer **124-I** due to the ring strain (Scheme 40).

**Scheme 41:**
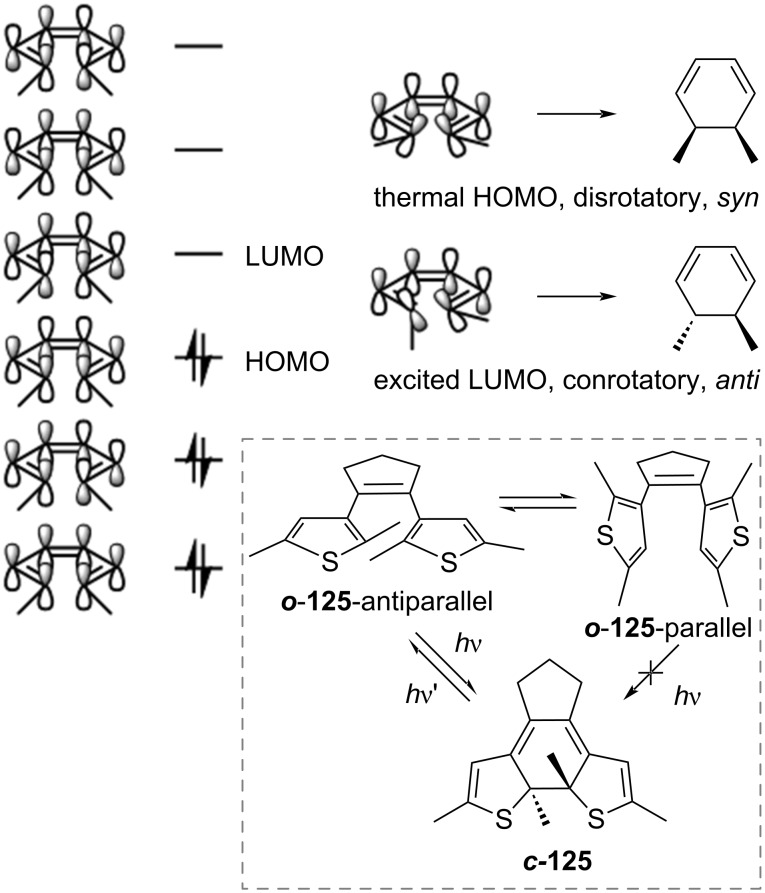
Molecular orbitals and mechanism of electrocyclisation for a 6π system.

According to the Woodward–Hoffmann rule [120], the excited state electrocyclisation of a 4*n* + 2 conjugated system proceeds in a conrotatory fashion. For steric reasons, the excited state ring closure is only possible when ***o*-125** is in antiparallel conformation (Scheme 41). However, open-ring diarylethenes usually consist of an equilibrium between a parallel conformation (not active) and antiparallel (active). For some examples, the equilibrium was also found to be affected by the polarity of the solvent: polar solvents could stabilise the parallel excited state because of the larger dipole moment [121]. To enrich the population of the antiparallel (and thus increasing the quantum yield of cyclisation), bulky substituents on the 2,2’ positions of benzothiophene were added [122], also the antiparallel conformation could be fixed by introduction of intramolecular interactions [123]; other solutions are the incorporation in polymers [124] or the confinement in supramolecular cages [125–126].

**Figure 21:**
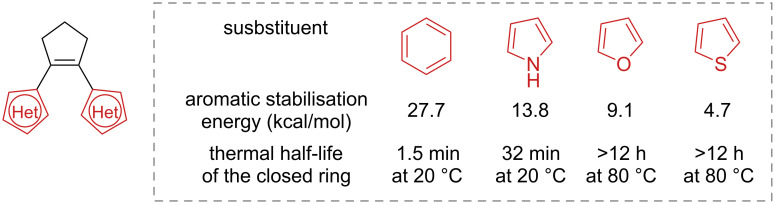
Aromatic stabilisation energy correlated with the thermal stability of the diarylethenes [127,129].

**Figure 22:**
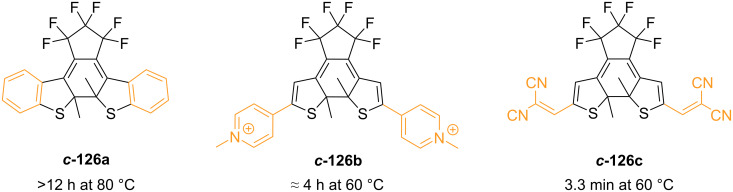
Half-lives of diarylethenes with increasing electron-withdrawing groups [128–129].

**Scheme 42:**
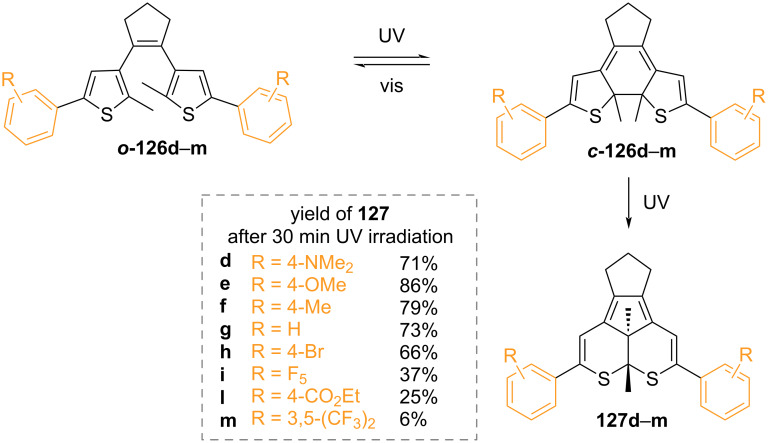
Photochemical degradation pathway promoted by electron-donating groups [130].

The thermal stability was found to depend primarily on the nature of the aryl rings: the high aromatic stabilisation energy of phenyl is correlated to a low activation energy barrier for the ground-state ring-opening [127]. Heterocycles with lower aromatic stabilisation energies are less susceptible to thermal ring-opening (Figure 21). The substituent nature of the aromatic groups was also demonstrated to influence the thermal stability. Strongly electron-withdrawing groups can weaken the photogenerated C–C bond, thus making the diarylethene thermally unstable, as in the case of ***c*-126b** and ***c*-126c** (Figure 22) [128–129]. Moreover, electron-withdrawing groups were also found to improve the fatigue resistance by avoiding the irreversible rearrangement **127** (Scheme 42) [130]. Besides influencing the thermal stability, the presence of substituents also increases the conjugation, typically giving a red-shifted closed-ring isomer. Electron-donating substituents were also found to have a strong red-shift effect and to increase the molar absorption coefficient of the closed form [131].

**Figure 23:**
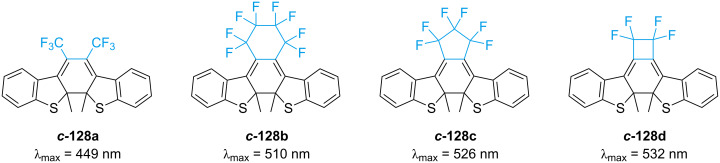
The diarylethenes studied by Hanazawa et al. [134]. Increased rigidity leads to bathochromic shift.

Regarding the ethene bridge, several cyclic and acyclic solutions have been extensively reported and reviewed [132–133]. Some of the most common bridges are pentene, perfluoropentene, maleic anhydride, and maleimide. An interesting study correlated the ring size of the bridge to the photophysical properties of four different perfluoroalkenylated diarylethene derivatives [134]. Unsurprisingly, the acyclic **128a** turned out to be the worst-performing in terms of fatigue resistance due to the competing *E*–*Z* photoisomerisation reaction. The fatigue resistance of the cyclic ones is comparable. However, a decrease in the ring size leads to more rigidity of the closed isomer, and thus major conservation of planarity, eventually resulting in a bathochromic shift of the closed-ring isomer (Figure 23). The absorption of the open-ring isomer is also influenced by the nature of the bridge: maleimide and maleic anhydride bridges are more red-shifted than perfluoroalkenes [129].

**Scheme 43:**
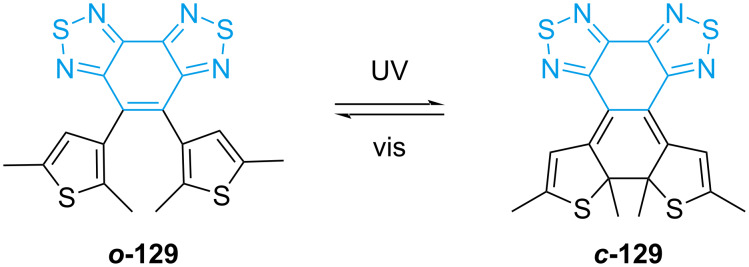
The dithienylethene synthesised by Nakatani's group [135].

Nakatani and co-workers synthesized dithienylethene **129** with a peculiar bridge that confers an extremely long thermal half-life and high resistance to fatigue (Scheme 43) [135]. The low aromaticity of the bridge results in an increased stabilisation upon ring closure, and the electron-withdrawing nature makes it more resistant to fatigue.

#### Synthesis

**Scheme 44:**
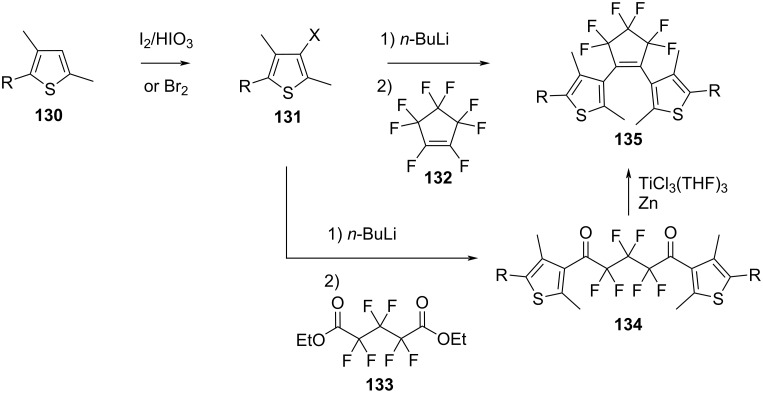
Synthesis of perfluoroalkylated diarylethenes.

The synthetic procedures for diarylethenes are various, and they strongly depend on the nature of the bridge. Typically, diarylethenes require some synthetic effort to be obtained, especially compared to azoheteroarenes or spiropyrans. Working with strong bases such as *n*-BuLi or using Pd-catalysed coupling methods requires handling under inert conditions by skilled synthetic organic chemists, which may not be suitable for every lab. Here, we list a few of the most common synthetic procedures. For less common derivatives, we refer the readers to an excellent review on the specific topic [132]. Perfluoroalkylated diarylethenes can be prepared by addition–elimination of perfluorocyclopentenes **132** [131] or perfluoroglutarates **133** [136] with a heteroarene **131** previously halogenated and treated with *n*-butyllithium. Cyclisation of the intermediate **134** to **135** is performed through McMurry coupling (Scheme 44). Variation of the reactant ratio gives access to symmetric and asymmetric diarylethenes.

**Scheme 45:**
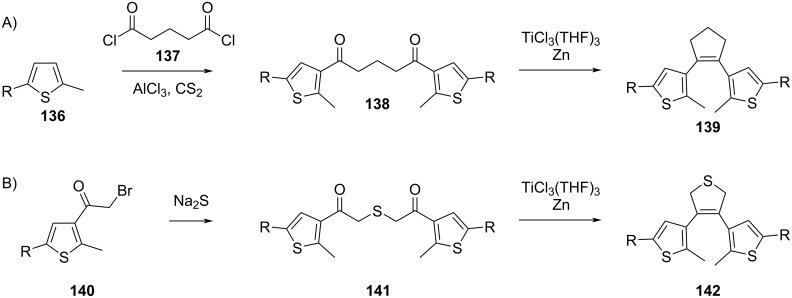
Synthesis of **139** and **142** via McMurry coupling.

The same strategy can be used for diarylcyclopentenes **139** (Scheme 45A) [137], starting from a Friedel–Crafts acylation of **136** with **137**. Homo-coupling of **140** (Scheme 45B) followed by McMurry coupling yields the 2,5-dihydrothiophene derivative **142** [138].

**Scheme 46:**
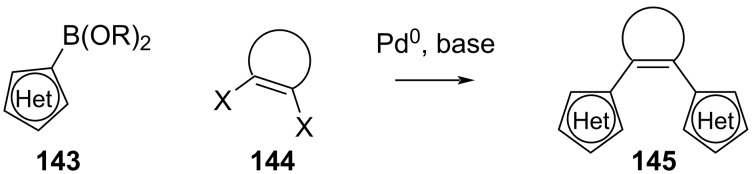
Synthesis of symmetrical derivatives **145** via Suzuki–Miyaura coupling.

Suzuki–Miyaura coupling (Scheme 46) with arylboronates **143** and dihaloalkenes **144** has also been reported for the synthesis of diarylethenes, however, the mono-coupling is not selective, thus it is more indicated for symmetrical derivatives [139–140].

**Scheme 47:**
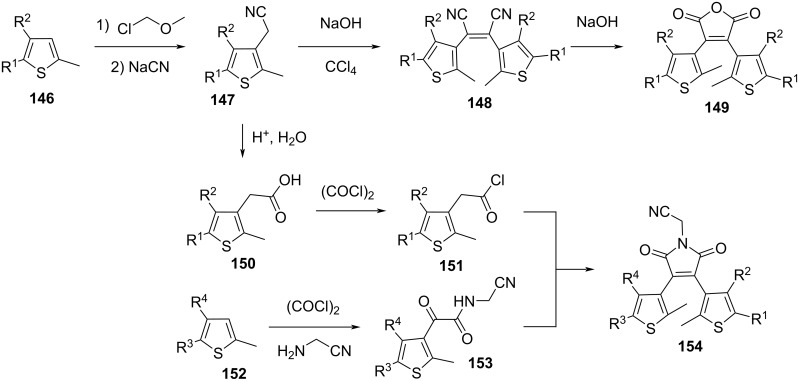
Synthesis of acyclic **148**, malonic anhydride **149**, and maleimide derivatives **154**.

Acyclic derivatives **148** can be synthesised by homo-coupling of **147**, while an extra step of basic hydrolysis can give the maleic anhydride derivative **149** (Scheme 47). Reaction of **152** with oxalyl chloride and aminoacetonitrile, followed by condensation with **151** yields maleimide derivatives **154** (Scheme 47) [129]. The R*^n^* substituents can be modified in a later stage through palladium coupling [141].

#### Examples

**Figure 24:**
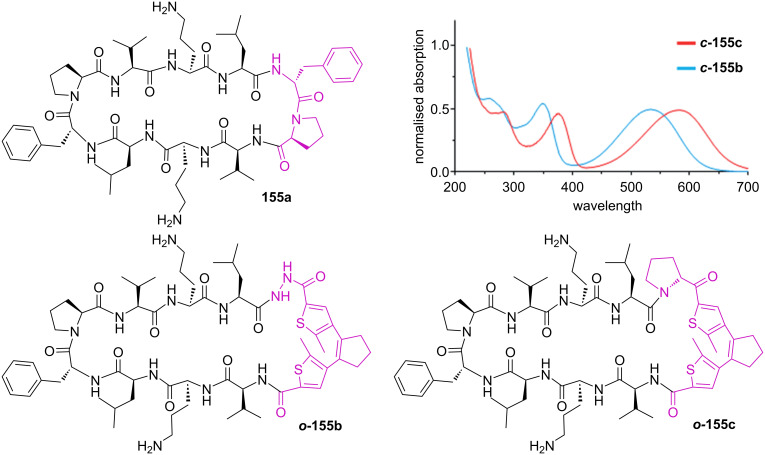
Gramicidin S (top left) and two of the modified diarylethene derivatives: first generation (bottom left) and second generation (bottom right). Top right: the difference in absorption of the closed isomers of the new photoswitchable derivatives. The upper right part of Figure 24 was adapted from [143], O. Babii et al., “Direct Photocontrol of Peptidomimetics: An Alternative to Oxygen-Dependent Photodynamic Cancer Therapy”, *Angew. Chem., Int. Ed.*, with permission from John Wiley and Sons. Copyright © 2016 WILEY-VCH Verlag GmbH & Co. KGaA, Weinheim. This content is not subject to CC BY 4.0.

Upon isomerisation, diarylethenes undergo both geometrical and electronic changes. The versatility contributed to the popularity of this photoswitch. An interesting application is found in the photoswitchable activity of a gramicidin S derivative where amino acids forming the β-hairpin of the cyclic peptide **155a** were substituted by a diarylethene moiety **155b** (Figure 24) [142]. The more rigid closed isomer ***c*-155b** influences the secondary structure of the gramicidin S derivatives, decreasing the antimicrobial activity, while the open form ***o*-155b** resembles more the original and keeps very similar activity. In a follow-up report, the hydrazide linker was substituted with a ketone, which provided ***c*-155c** with a red-shifted absorption spectrum [143].

**Scheme 48:**
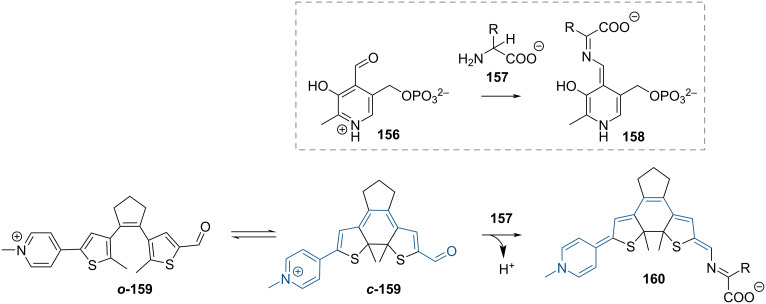
Pyridoxal 5'-phosphate and its reaction with an amino acid (top). The analogous dithienylethene derivative (bottom).

The pyridoxal 5´-phosphate cofactor mimic reported by Branda and co-workers is an elegant example of photoswitchable electronic properties [144]. The diarylethene mimic ***o*-159** (Scheme 48) is inactive because the electron-withdrawing pyridinium ion is insulated from the aldehyde reactive site. Upon ring closure, the pyridinium becomes conjugated with the aldehyde, which can react with an amino acid, yielding **160**. The difference in activity between the isomers was measured by monitoring the racemisation rate of enantiopure ʟ-alanine.

### Fulgides

**Figure 25:**
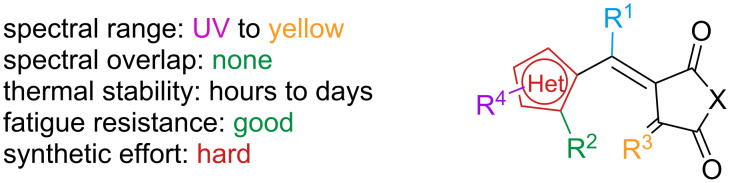
Fulgides.

Fulgides are a class of photoswitches first reported by Stobbe in 1905, which are named after the Latin word *fulgere* (to shine) due to their bright colours [145] (Figure 25).

**Scheme 49:**
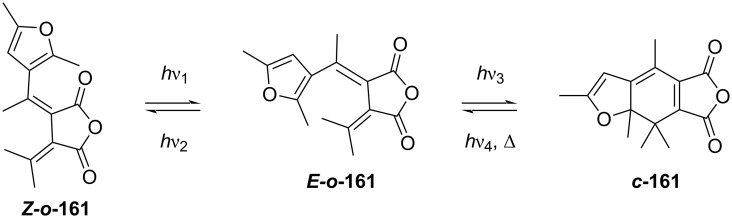
The three isomers of fulgides.

The name refers to the original bismethylenesuccinic anhydrides in Scheme 49, while more derivatives have been subsequently synthesised, among which we find fulgimides, fulgenolides, and fulgenates. They are thermally stable, resistant to fatigue, and have fast response to irradiation. As in the case of diarylethene, fulgides can also undergo *E*–*Z* isomerisation (Scheme 49). The *Z*-isomer ***Z-o*-161** cannot undergo ring closure to ***c*-161** without converting to the *E*-isomer first ***E-o*-161**.

**Scheme 50:**
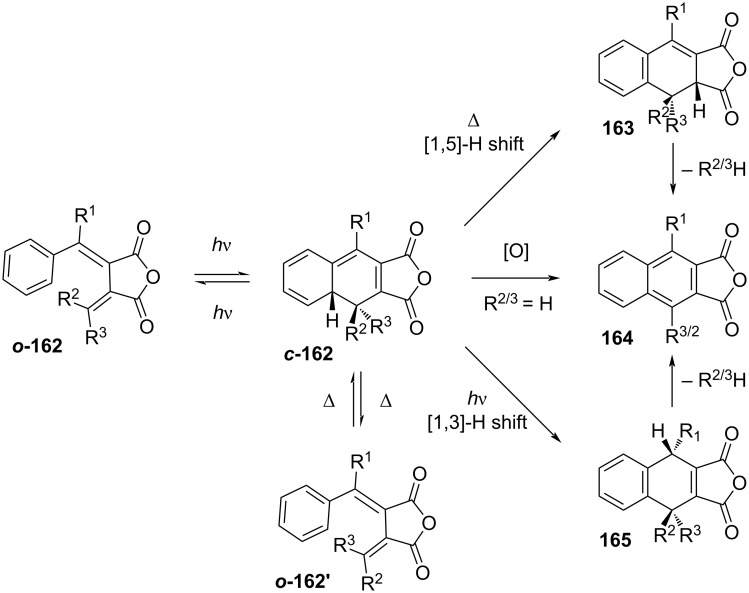
Thermal and photochemical side products of unsubstituted fulgide [150].

Similarly to what was already discussed for diarylethenes, the excited-state ring closure is conrotatory (see Scheme 41). In order to avoid irreversible oxidation of the closed isomer, all the hydrogen atoms of the hexatriene backbone must be substituted for bulkier groups. It was reported that the presence of hydrogen substituents generates irreversible side products of elimination (**164**), disrotatory ring-opening (***o*-162’**), thermal (**163**), and photoinduced sigmatropic hydrogen shifts (**165**) (Scheme 50) [146–148]. Moreover, it was reported that substitution of the phenyl ring for a furan would decrease the spectral overlap between the open and the closed isomer, thus improving the PSS [149].

**Figure 26:**
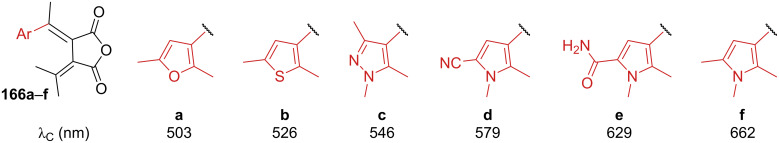
Maximum absorption λ_c_ of the closed isomer compared with the nature of the aromatic ring and the substitution pattern (selected examples). λ_c_ was determined on poly(methyl methacrylate) film.

Many other heteroaromatics have been employed ever since. A systematic study showed that the absorption maximum red-shifts with increasing electron-donating character of the heteroaromatic ring and of the substituents, while electron-withdrawing substituents cause a hypsochromic shift (Figure 26). The effect is stronger in the closed isomer [150–151].

**Scheme 51:**
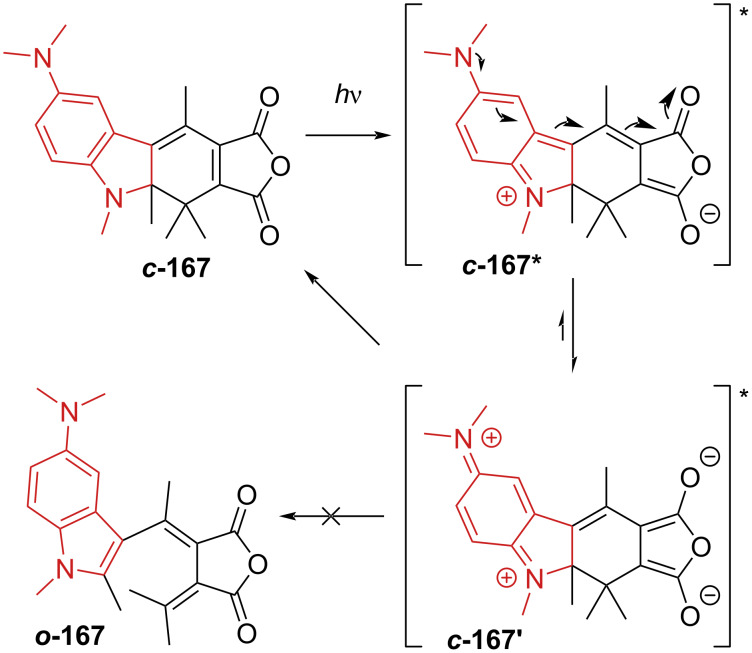
Possible rearrangement of the excited state of 5-dimethylaminoindolylfulgide [153].

Some fused heterocycles and sterically hindered substituents were also reported. However, it was proposed that such substituents lead to a loss in coplanarity in the closed form and/or a higher stability of the heteroaromatic, thus lowering the quantum yield of ring closure and the ratio of *C* to *E*-isomer at the PSS. A methoxy substituent in 6-position of an indolinefulgide was proven to increase the absorption coefficient of the closed form [152]. Another study reports a bathochromic shift for an indolinefulgide with methoxy substitution in the 5-position and an unusually low ring-opening quantum yield for **167** with a dimethylamino substituent [153]. The proposed hypothesis is that with such a strong electron-donating group, an electron displacement in the excited state *c*-167* occurs (Scheme 51) that eventually returns to ***c*-167**. This is in accordance with the general observation that electron-withdrawing groups improve the ring-opening process and disfavour the ring closure, while for the electron-donating groups, it works the other way round [151]. A similar explanation can be used to justify the solvent dependence on the ring-opening quantum yield of thionylfulgide observed in Tomoda’s group: The quantum yield of ring-opening was lower in ethyl acetate than toluene, thanks to the better stabilisation of the polar excited state by the more polar solvent [154].

**Figure 27:**
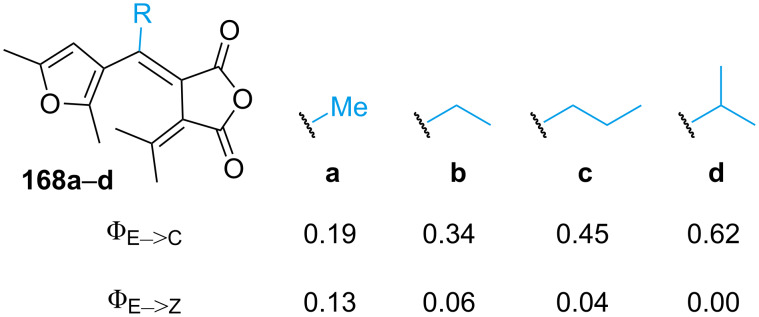
Quantum yields of ring closure (Φ_E→C_) and *E–Z* isomerisation (Φ_E→Z_) correlated with the increasing steric bulkiness of substituent R.

As already mentioned, the open form of fulgide can exist as *E-* and *Z*-isomer, for which the former one is the only one active for the 6π-electrocyclisation. The effect of bulky substituents in **168** was studied, and it was demonstrated to inhibit dramatically the *E*–*Z* isomerisation due to the increasing steric hindrance (Figure 27) [155]. A report of indolylfulgide with a trifluoromethyl group instead of methyl showed higher thermal stability of the closed form and higher resistance to fatigue. The authors explain that this is due to the absence of allylic hydrogens that can undergo abstraction in the excited state [156].

**Scheme 52:**
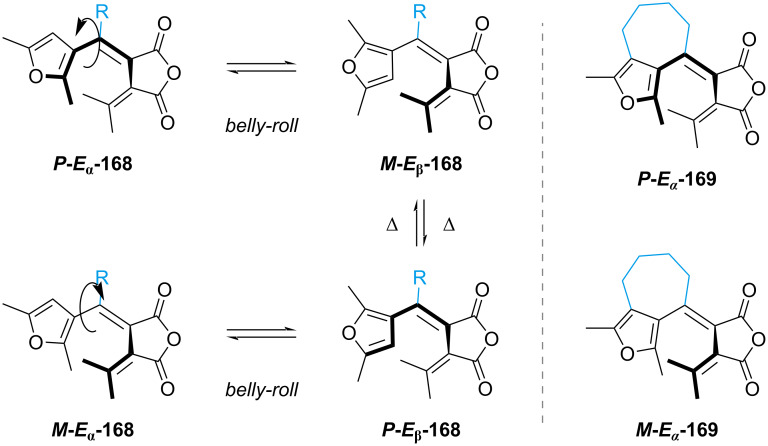
Active (*E*_α_) and inactive (*E*_β_) conformers (left) and the bicyclic sterically blocked fulgide **169** (right).

It must be noted that, given the helical nature of the open fulgide, it presents two couples of enantiomers (right-helical *P* and left-helical *M*), which can thermally interconvert [157]. Besides the photoinduced *E–Z* isomerisation, the thermal rotation at room temperature of the furyl moiety (*belly-roll*) leads to the *E*_β_ conformer, which is unable to cyclise (Scheme 52, left). Changing the furan to a benzofuran shifts the equilibrium towards the active *E*_α_ conformer, however, to fully suppress *E–Z* isomerisation and the thermal isomerisation to the inactive *E*_β_ conformer, the bicyclic derivative **169** was reported (Scheme 52, right) [158].

**Scheme 53:**
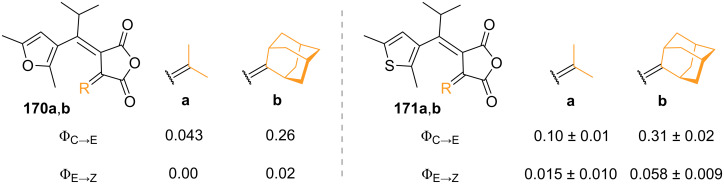
Quantum yield of ring-opening (Φ_C→E_) and *E–Z* isomerisation (Φ_E→Z_) for different substitution patterns.

Furylfulgide **170b** [159] and thiophenfulgide **171b** [160] with an adamantyl group were correlated with their isopropyl derivatives, and they both show a net increase in the ring-opening quantum yield, however, associated with a slight increase in *E–Z* isomerisation (Scheme 53).

Fulgimides have in general very similar properties to fulgides, but they offer a few advantages like improved resistance to hydrolysis in protic solvents and easy functionalisation on the maleimide nitrogen (see next section "Synthesis"). Thus, they are preferred, especially for biological applications [161]. Fulgenates have diesters instead of the anhydride. They are also easier to functionalise and are resistant to hydrolysis. However, the freedom of rotation around the bond between the two esters makes them less appealing in terms of photophysical properties. Substitution of the maleic anhydride with a lactone (fulgenolides) or with other heteroatoms was also reported [151].

#### Synthesis

**Scheme 54:**
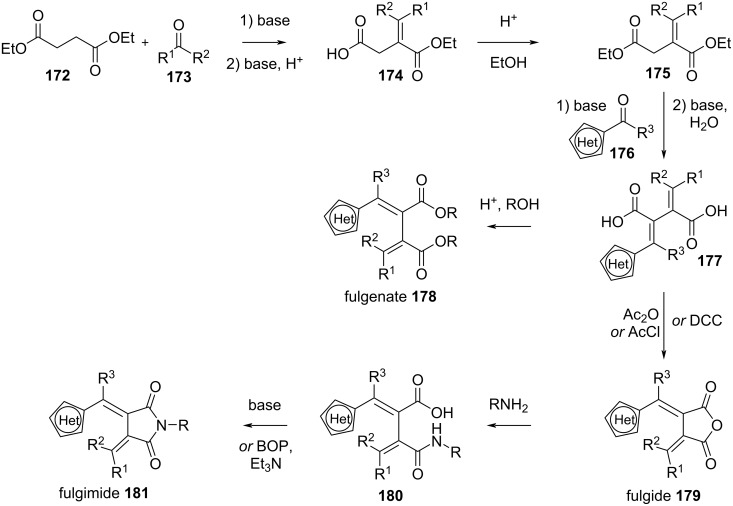
Stobbe condensation pathway for the synthesis of fulgides **179**, fulgimides **181** and fulgenates **178**.

Fulgides, fulgenates, and fulgimides require some synthetic effort, especially when compared to azoheteroarenes and spiropyrans. The classic synthetic pathway [146], shown in Scheme 54, is through a Stobbe condensation of **172** and **173** followed by esterification, a second Stobbe condensation of **175** and **176** and saponification. At this point, through acidic esterification of **177** one can obtain fulgenates **178**. Fulgides **179** are obtained through condensation with acetic anhydride, acetyl chloride, or dicyclohexylcarbodiimide (DCC) [162]. Further amidation and condensation by base or by means of coupling agents [163] yields fulgimides **181**.

**Scheme 55:**
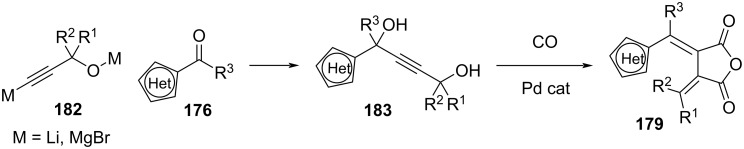
Alternative synthesis of fulgides through Pd-catalysed carbonylation.

Due to the low yields of the Stobbe condensation, another pathway was designed specifically for sterically demanding substituents (Scheme 55) [164–165]. Reaction of the lithiated alkyne **182** with the heteroarylketone **176** yields the precursor **183**, which is then carbonylated via palladium catalysis.

**Scheme 56:**
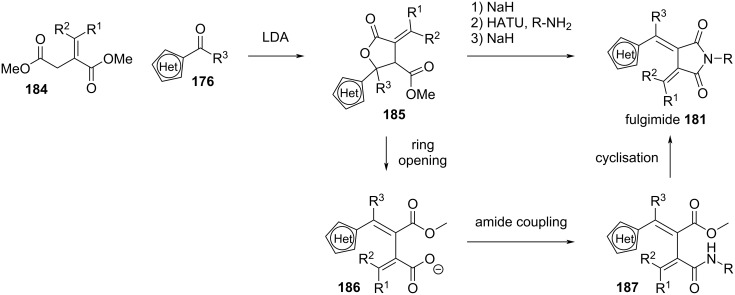
Optimised synthesis of fulgimides [166].

Slanina and co-workers recently developed an improved synthetic strategy to yield fulgimides via a sequential one-pot procedure (Scheme 56). For this purpose, they start with cyclic lactone **185** which under basic conditions opens to intermediate **186**. Treatment with HATU and an amine source converts the carboxylic acid moiety to the corresponding amide **187** which then can cyclise again to the target fulgimide **181** upon treatment with NaH. This synthesis is not only faster compared to classical procedures, but also does not require the purification of intermediates, overall increasing the accessibility of fulgimides [166].

#### Examples

**Scheme 57:**
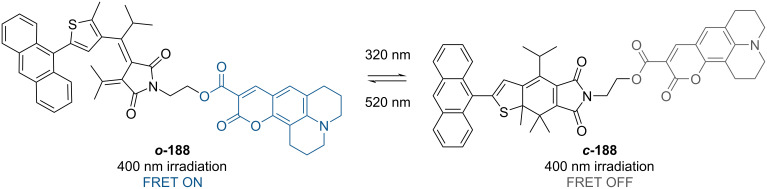
Photoswitchable FRET with a fulgimide photoswitch [167].

Fulgimide **188** was used as a bridge between a fluorescence resonance energy transfer (FRET) donor (anthracene) and an acceptor (coumarin) (Scheme 57). The open form ***o*-188** allows intramolecular energy transfer between anthracene and coumarin, resulting in fluorescence emission of the coumarin. However, in the closed isomer ***c*-188** the fluorescence is quenched. The closed isomer acts like an energy trap, dissipating the energy through radiationless pathways and inhibiting the FRET [167].

**Scheme 58:**
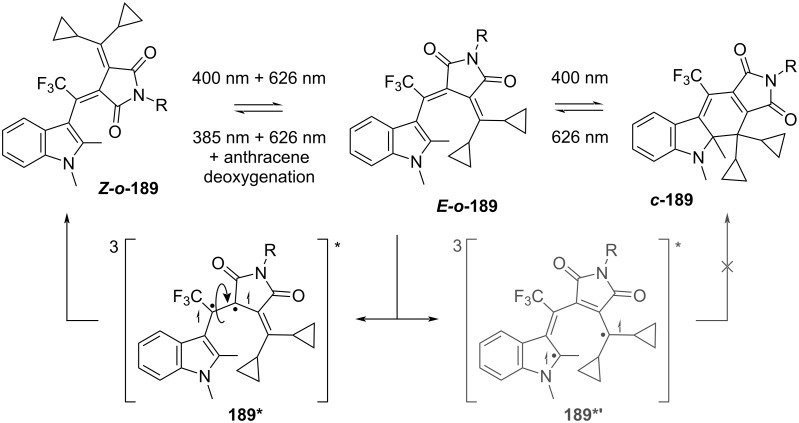
Three-state fulgimide strategy by Slanina's group.

Slanina and co-workers developed a three-state fulgimide photoswitch (Scheme 58). Irradiation of ***Z-o-*189** with UV light leads to ***c*-189**, while a combination of UV and red light leads to the open ***E-o-*189**. The least stable *Z*-isomer can be accessed quantitatively through triplet-state sensitization (with addition of anthracene as a sensitizer and by removal of oxygen from the solution) and dual irradiation with UV and red light [166].

### Spiropyrans

**Figure 28:**
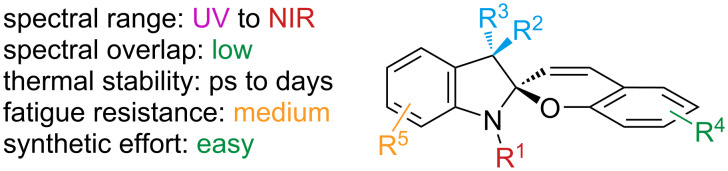
Spiropyrans.

Another important and versatile class of photoswitches are spiropyrans, named after their spirocyclic carbon atom centrally located in the molecular structure, which plays a crucial role in switching [168] (Figure 28).

**Scheme 59:**
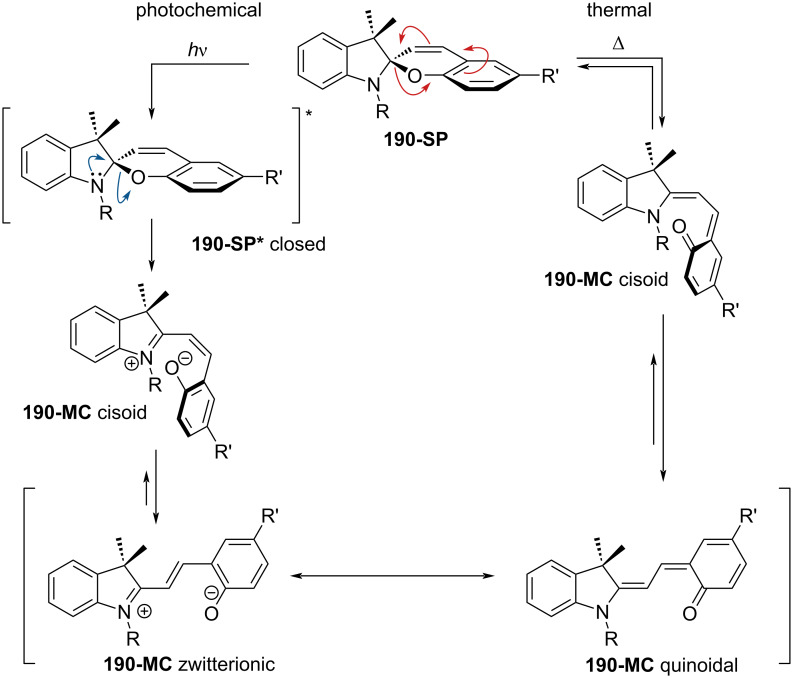
Photochemical (left) and thermal (right) ring-opening mechanisms for an exemplary spiropyran with arbitrary substituents R and R’. The blue arrows indicate a photochemical cleavage of the C_spiro_–O bond, while the red arrows represent a thermal 6π-electrocyclic ring-opening [169].

They are well known for their drastic change in geometry and polarity when switched from the closed spiropyran form (SP) to the open merocyanine isomer (MC) [169]. Besides that, they show a broad spectrum of responsiveness to external influences other than light, reaching from solvato- to acidochromism, high quantum efficiencies for direct and back photoinitiated rearrangements, and very high cross sections of two-photon light absorption of ring-opened and ring-closed forms [170–172]. Apart from classical spiropyrans, there are the related classes of spirooxazines and spironaphthopyrans. As they share some common features, they are also briefly discussed within this section. The first feature to be described was their thermochromic behaviour [173]. Some years later, Fischer and Hirshberg observed that SPs are not only thermochromic but by irradiating with UV light, one can generate the same colourful species as upon heating [174]. For both the thermal and photochemical cases, decolourisation was observed after a certain time, indicating that the switching process is, in principle, reversible. Scheme 59 shows the ring-opening mechanisms for the photochemical and thermal cases. While in the photochemical case, the C_spiro_–O bond gets cleaved in the excited state 190-SP*, the thermal process can be described as 6π-electrocyclic ring-opening of the pyran ring. In both cases, the open **190-MC** isomer is formed, which can be zwitterionic or quinoidal and thermally re-isomerises back to the closed spiropyran form [169].

**Figure 29:**
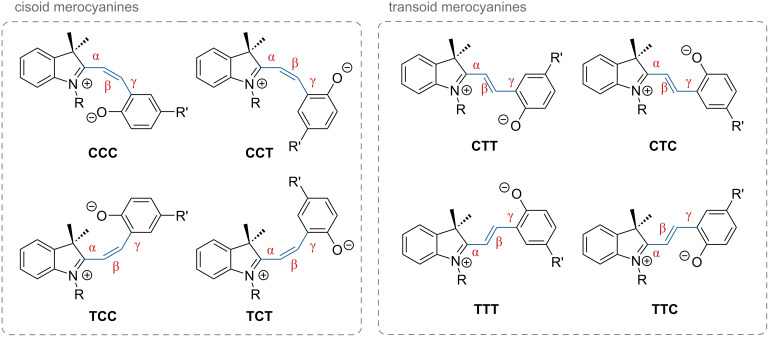
Eight possible isomers of the open merocyanine according to the *E*/*Z* configurations of the bonds highlighted in blue (α, β, and γ). The orientation of the centrally located double bond (β) is used for classification into the two groups cisoid-merocyanines (β → *Z* configuration) and transoid-merocyanines (β → *E* configuration). The abbreviations of the isomers are composed of a sequence of the orientations of the bonds α, β, and γ (e.g. TCT = trans–cisoid–trans) [170].

A total of eight different MC isomers can be formed (Figure 29), the TTC and TTT being the most stable ones [170,175–176]. In most organic solvents, the rather non-polar closed SP form is predominant under thermal conditions. Upon irradiation with UV light (or NIR light in the case of two-photon absorption) [171,175–176], one can switch it to the ring-opened merocyanine isomer, which thermally re-isomerises back to the closed SP form with a particular lifetime (depending on the photoswitch itself, the temperature, and the conditions of the system) [177].

This low energy barrier for re-isomerisation classifies spiropyrans as a T-type photoswitch [3]. Interestingly, since the colourless SP gets interconverted to the intensely coloured MC form by light, positive (or direct) photochromism can be observed in organic solvents [2]. In aqueous conditions, however, the open merocyanine isomer is sufficiently stabilised due to its zwitterionic character (dipole moment µ = 14–18 D, in contrast with µ ≈ 4 D for SP) [169,178]. As a result, a noteworthy amount of MC is present at thermal equilibrium, which can be interconverted to the colourless SP form upon irradiation with light in the visible regime. In this case, the spiropyran photoswitch shows negative (or inverse) photochromism [3,179]. The degree of stabilisation of either MC or SP depends on the polarity of the solvent and on the substitution pattern [2,180–181].

**Scheme 60:**
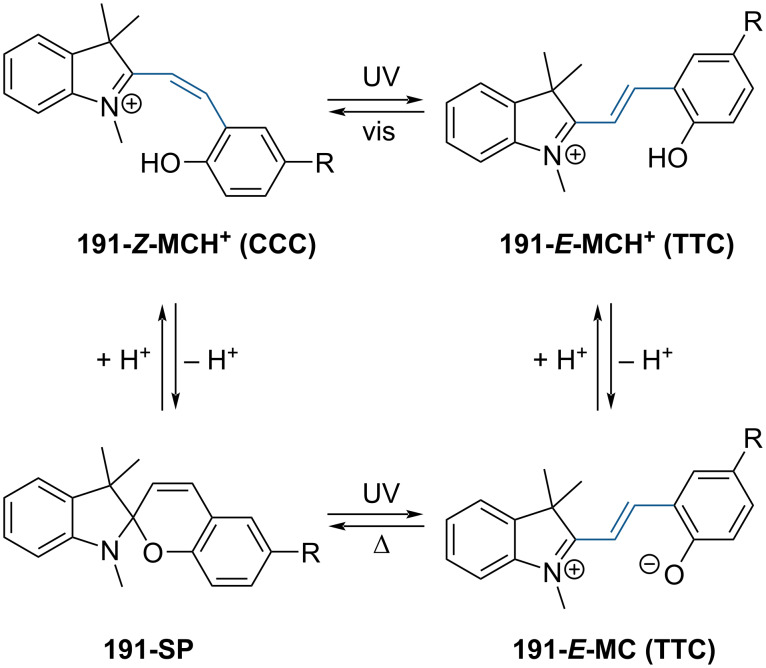
pH-Controlled photoisomerisation between the closed spiropyran **191-SP** and the open *E*-merocyanine **191-*E*-MC** as well as the protonated *E*-merocyanine **191-*E*-MCH^+^** and the *Z*-merocyanine **191-*Z*-MCH^+^**. Both *E*-merocyanines are present in the most stable trans–transoid–cis (TTC) configuration, and the *Z*-merocyanine shows the cis–cisoid–cis (CCC) geometry (R = H, NO_2_). Note that **191-*Z*-MCH^+^** can only switch back to **191-SP** upon deprotonation, which enables pure *E*/*Z*-photoisomerisation when the medium is strongly acidic [175].

Besides that, the MC form can be reversibly protonated in aqueous conditions, as illustrated in Scheme 60. Thereby, the absorbance spectra of the non-protonated **191-MC** and the protonated merocyanine **191-MCH^+^** differ notably, and a pH-dependent colour change can be observed, referred to as *acidochromism.* The degree of protonation also plays an essential role in the ring-closing to **191-SP** since only the non-protonated merocyanine isomers **191-MC** can convert to **191-SP**. In contrast, for the protonated ones **191-MCH^+^**, this process is not feasible anymore [175]. Hence, this opens the opportunity for pH-gated *E*/*Z*-isomerisation of the **191-MCH^+^** isomers by visible or UV light under very acidic conditions without converting them to **191-SP** [175]. Further, by the addition of acid with its p*K*_a_ higher than the p*K*_a_ of **191-*Z*-MCH^+^** and lower than the one of **191-*E*-MCH^+^**, one can create a photoacid generator. Photochemical conversion of **191-*E*-MCH^+^** to **191-*Z*-MCH^+^** re-protonates the used acid, followed by ring-closure to **191-SP**, which can again be photochemically opened to form the **191-*E*-MC**. Re-protonation to **191-*E*-MCH^+^** by the present acid closes the cycle [175].

**Scheme 61:**
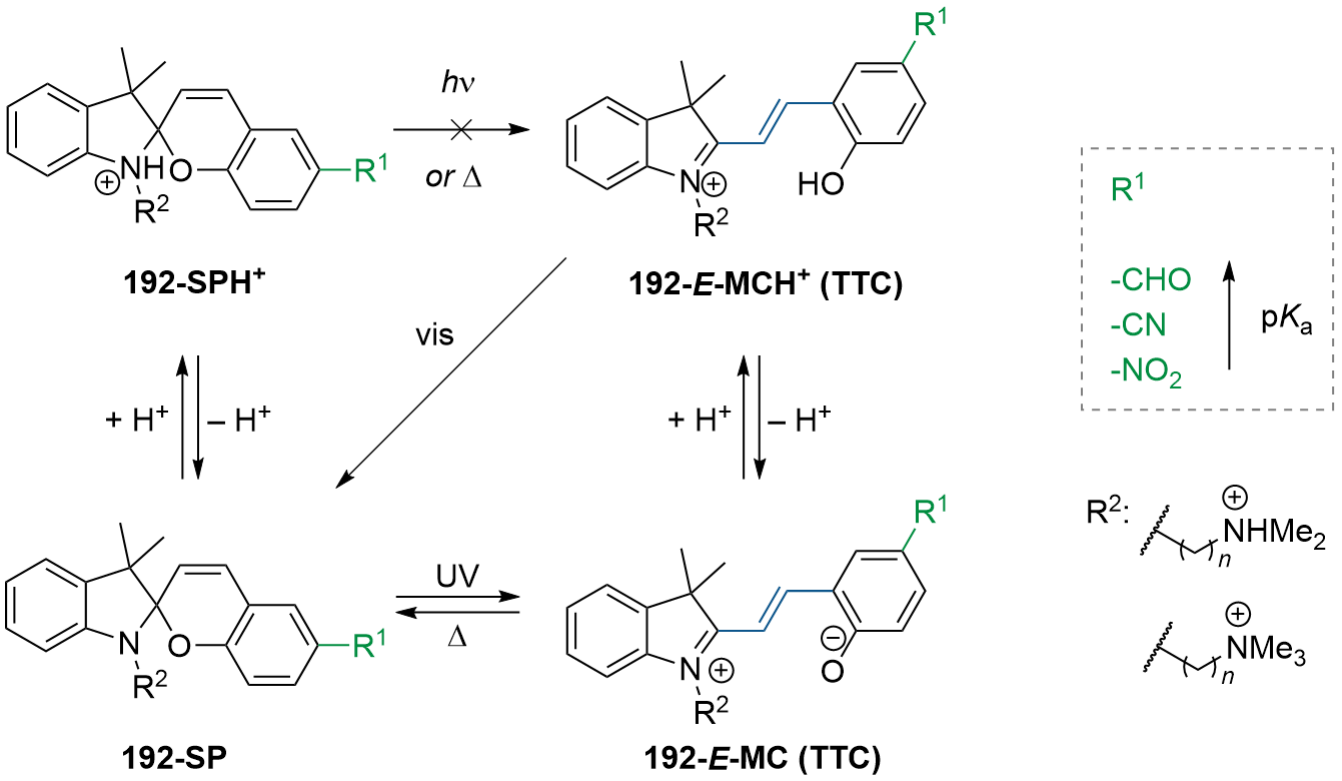
Behaviour of spiropyran in water buffer according to Andréasson and co-workers [180]. **192-SP** in an aqueous medium can only convert to **192-MC** when it is not protonated. Box: effect of the R^1^ group on the p*K*_a_ of the phenol group. The higher the p*K*_a_, the more shifted the equilibrium towards the protonated **192-MCH^+^**.

A study by Andréasson and co-workers reported that substitution at R^2^ with alkyl chains bearing a terminal amine group allows photoswitchable behaviour of spiropyrans in aqueous buffer also at acidic conditions (Scheme 61) [180]. The acidity of the phenol can be fine-tuned by the choice of R^1^ (Scheme 61, box), with electron-withdrawing groups stabilising the **192-MC** at lower pH [180,182]. The positively charged substituent probably avoids further protonation of the indoline nitrogen in the **192-SP** isomer, which would slow down isomerisation. Indeed, the authors attribute the lower rate to the presence of the species **192-SPH^+^**, which cannot isomerise to **192-MC** directly but only through equilibrium with **192-SP** (Scheme 61) [180]. It must be noted that the observations are done in an aqueous buffer and that the solvent and the environment play a crucial role, especially for this photoswitch class. It is noteworthy also that **192-*E*-MCH^+^** in aqueous media switches to **192-SP** with visible light upon proton release, while **192-*E*-MC** switches to **192-SP** thermally [183]. For a detailed analysis of spiropyrans in water, we invite you to read the excellent review by Berton and Pezzato [183].

**Scheme 62:**
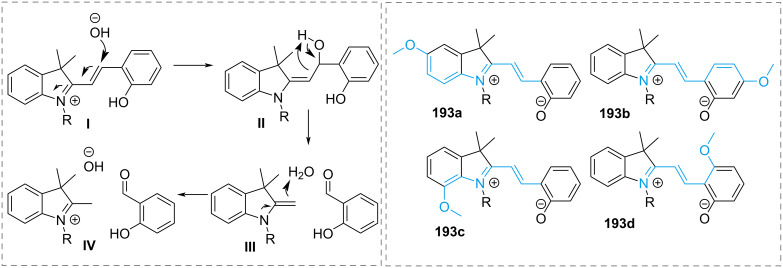
(left box) Proposed mechanism of basic hydrolysis of MC [184]. (right box) Introduction of electron-donating groups to decrease the electrophilicity of the double bond [184–185].

While the pH-responsive behaviour of spiropyrans, in principle, enables their use for various applications where pH plays a crucial role, one also has to deal with their tendency to hydrolyse, especially in aqueous media. It was found that the phenolic oxygen atom of the non-protonated merocyanine (MC) is crucial for the hydrolysation to proceed, coordinating to water molecules acting as nucleophiles. Changing the p*K*_a_ of the phenol moiety slows down the hydrolysis rate, but only apparently, as it will also shift the thermal equilibrium towards the spiropyran form [180]. A later report proposes a different hydrolysis mechanism that rules out the role of phenolate (Scheme 62, left) [184]. A strategy to slow down the nucleophilic attack of water is to introduce electron-donating groups directly conjugated with the ene–iminium core in order to decrease the electrophilicity of the system (Scheme 62, right) [183–185].

**Scheme 63:**
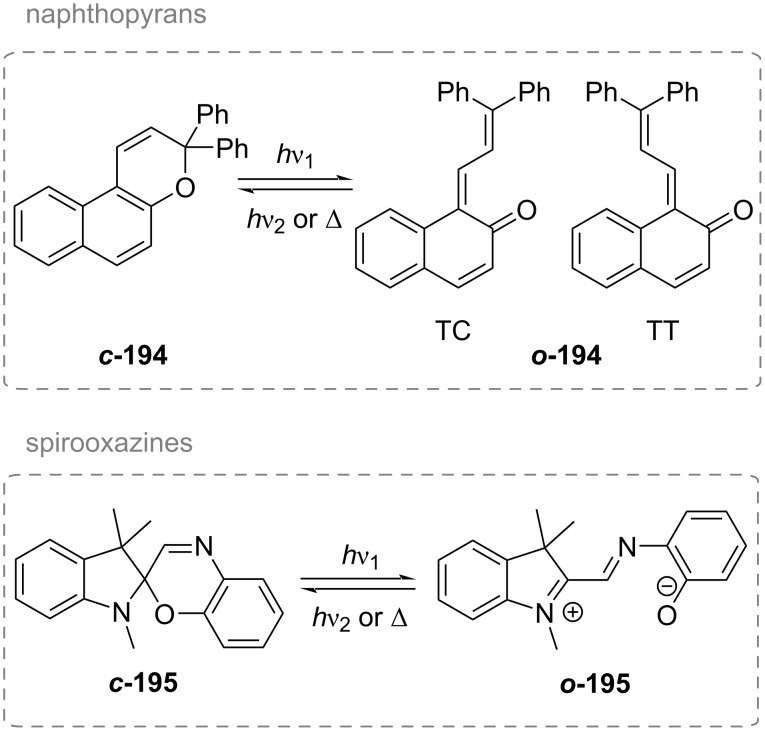
Photochemical interconversion of naphthopyran **194** (top) and spirooxazine **195** (bottom) photoswitches from the closed to the open forms and thermal or light-induced ring closure. TC = transoid–cis, TT = transoid–trans isomers of the open naphthopyran species [188–189].

Louie and co-workers investigated the effects different substitution patterns have on the photophysical behaviour of spiropyrans and spirooxazines more closely [178]. Therefore, they tuned the electron-withdrawing effect on the chromene and indoline moiety by combinations various functional groups at position 5’ on the methylene indoline moiety and position 6 on the chromene unit. While for 5’-OMe spiropyrans the photoconversion process SP → MC is drastically enhanced the more electron-withdrawing the substituent on the chromene unit is, there is no direct trend for varying substituents on the indoline moiety, given a fixed NO_2_ group on the 6-position of the chromene unit. By increasing the electron-withdrawing effect even more by incorporating a positively charged tertiary nitrogen atom in the chromene ring, the photochemical response was enhanced, together with the thermal stability. Interestingly, spirothiopyrans were also reported, where the oxygen atom is substituted by a sulphur atom, resulting in a strong red-shift of the open form up to the infrared region. However, the compound shows photochromic behaviour only below 0 °C [186]. It was found that by simple addition of a nitro group in *p*-position to the sulphur, the open form was stable up until 80 °C, and the absorption could be further tuned by introducing several substituents in various positions [187]. Similar to spiropyrans, naphthopyrans **194** can also be switched between closed and open forms TC (transoid–cis) and TT (transoid–trans) upon irradiation (Scheme 63, top). The metastable TC isomer thermally reverts back to the closed form within seconds to minutes, while the TT isomer takes minutes to hours for the ring closure. Like in the case of spiropyrans, the behaviour of thermal re-isomerisation renders naphthopyrans T-type photoswitches, and their absorption properties, as well as their lifetimes, can be tuned with different substituents [188]. This subclass, however, does not possess zwitterionic character in either form. A further class of spiropyran-related photoswitches are spirooxazines **195**. They contain a nitrogen atom in the 6-membered ring of the spiro junction and can also undergo reversible cyclisation. Driven by light, the C_spiro_–O bond gets cleaved, leading to the open form (Scheme 63, bottom), which can be switched back to the closed one either by light of another wavelength or thermally. Spironaphtooxazines, analogues of spirooxazines, have been more extensively studied due to synthetic availability [189].

#### Synthesis

**Scheme 64:**
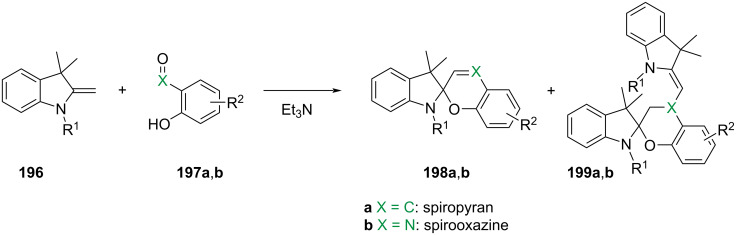
Synthesis of spiropyrans and spirooxazines **198** and the dicondensation by-product **199**.

**Scheme 65:**
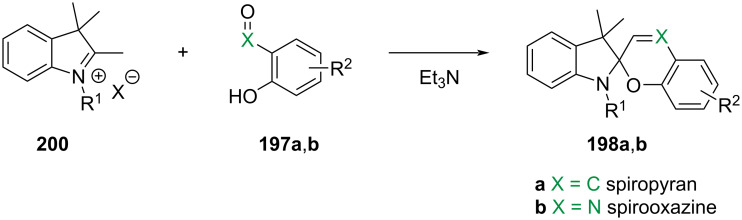
Alternative synthesis of spiropyrans and spirooxazines with indolenylium salt **200**.

One big advantage of spiropyrans is their synthetic accessibility. Compared to other photoswitch classes presented in this review, the synthetic effort to obtain spiropyrans is relatively low. The most common synthesis of spiropyran is the condensation of a Fischer indoline base **196** with a salicylaldehyde **197a**, in basic media, as shown in Scheme 64 [170]. However, this synthetic route can give a dicondensation by-product **199**, due to the high reactivity of indoline. To avoid this, it is possible to use a less reactive quaternary indolenylium salt **200** (Scheme 65) [190]. The same synthetic pathway can be used for the synthesis of spirooxazine **198b**, substituting the salicyladehyde **197a** with *o*-hydroxynitrosobenzene **197b** [189].

**Scheme 66:**
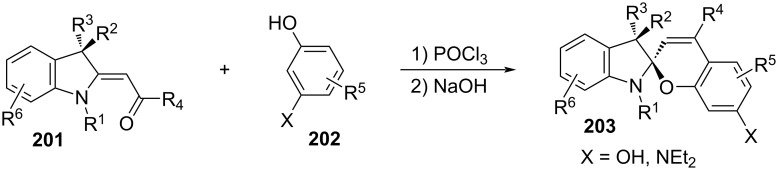
Synthesis of 4’-substituted spiropyrans **203** by condensation of an acylated methylene indoline **201** with a phenol derivative **202** (e.g., resorcin). R^1^–R^6^ are arbitrary alkyl, aryl, or heteroaryl substituents [190].

Another way to obtain spiropyrans is the reaction of an acylated methylene indoline species **201** with a phenol **202** upon the addition of POCl_3_ and subsequent treatment with NaOH. By that, it is possible to install a substituent R^4^ (Scheme 66).

**Scheme 67:**
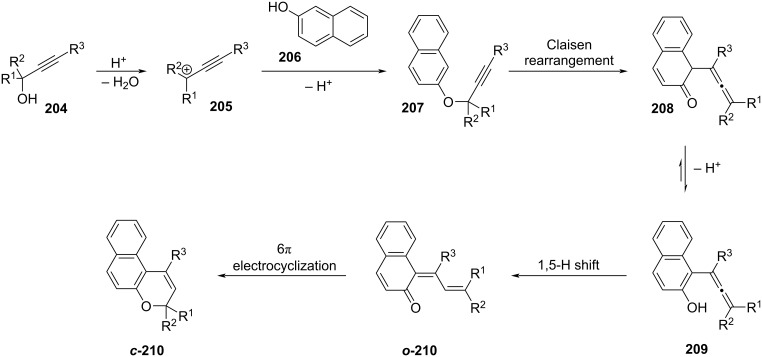
Synthesis of spironaphthopyrans **210** by acid-catalysed condensation of naphthols and diarylpropargyl alcohols [191].

Naphtopyrans **210** can be synthesised by acid-catalysed condensation of naphthols **206** and diarylpropargyl alcohols **204**. The reaction proceeds in one pot via multiple steps, which are shown in Scheme 67. To facilitate the electrocyclisation step in cases where ***o*-210** is stabilised, it is recommended to irradiate the reaction mixture with visible light [191].

#### Examples

**Scheme 68:**
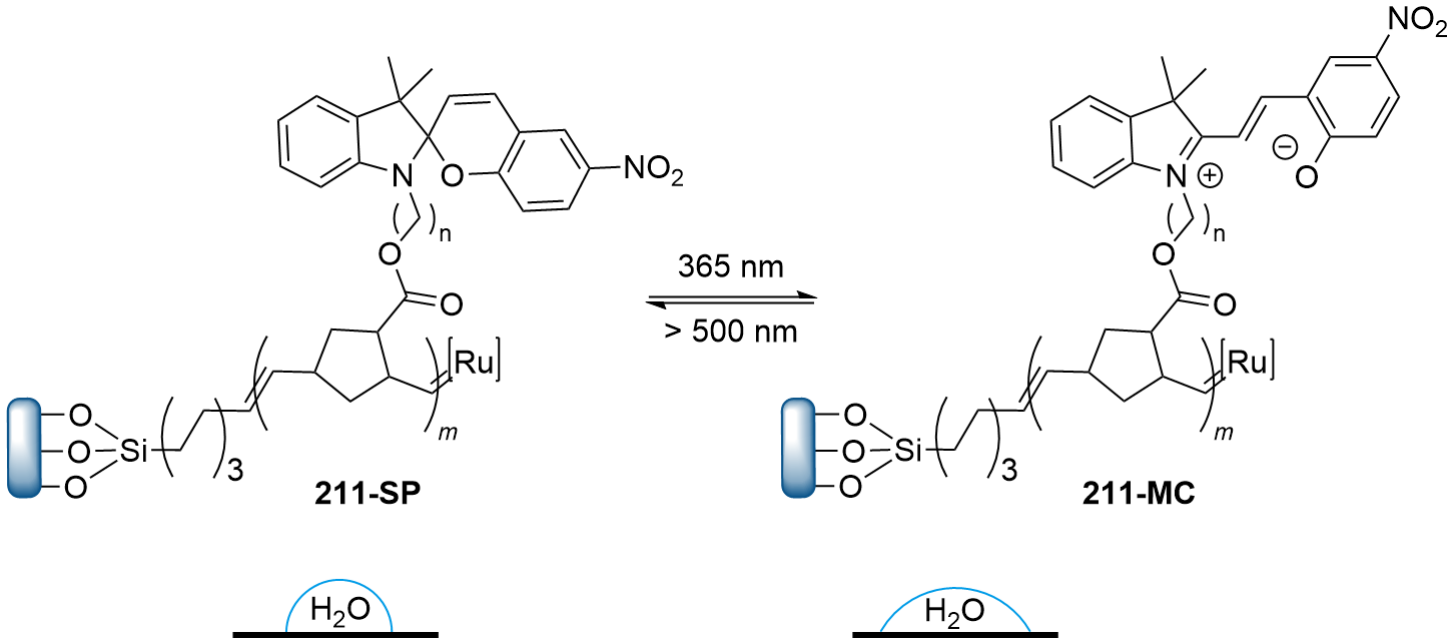
Photoswitchable surface wettability [194].

Spiropyrans have been extensively used in materials science to design smart materials [192]. For example, the decoration of polymers with spiropyrans can alter their macroscopic properties, such as surface wettability, conductivity, and mechanical properties with light [193]. A spiropyran polymer brush **211** induced a difference in surface wettability upon irradiation, due to the major affinity of the zwitterionic **211-MC** to water (Scheme 68) [194].

Spiropyrans have also demonstrated applicability in the biological environment: Feringa and co-workers were able to modify the naturally occurring channel protein MscL (mechanosensitive channel of large conductance) from *Escherichia coli* by covalently connecting a spiropyran photoswitch to the cysteine residues of the channel protein. Photochemical ring opening of the spiropyran with 366 nm light leads to weakening of the hydrophobic forces that keep the channel in its closed state, resulting in channel opening, while visible-light irradiation restores the initial state [195].

## Conclusion

**Figure 30:**
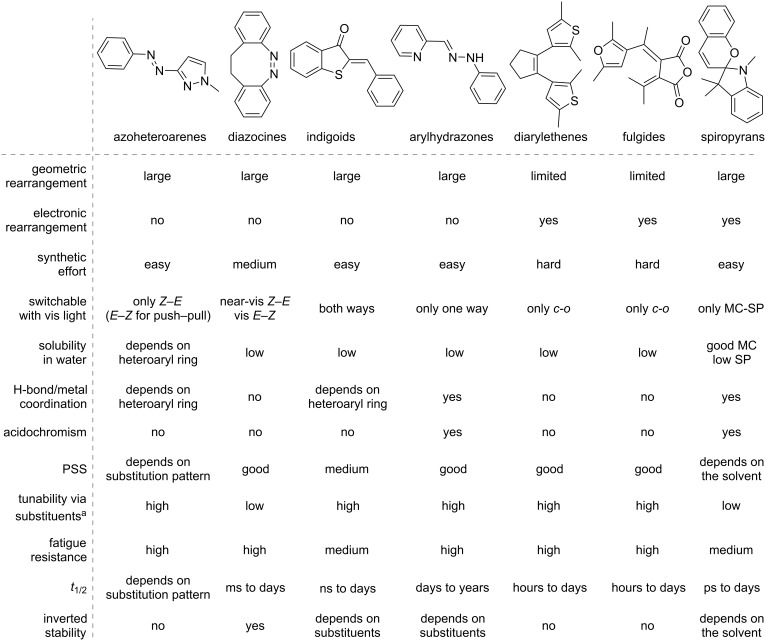
Some guiding principles for the choice of the most suitable photoswitch. Note that this guide is very general, and the properties can be tailored by choosing the right substitution pattern, concentration and medium, as discussed throughout the review. ^a^Takes into account the effect of the substituent and the number of different positions that can bear substituents.

In the field of photoswitches, many alternatives to azobenzenes remain underrepresented. While these alternatives may not be as well-known, they possess intriguing photophysical and mechanical properties. This review describes the synthesis, structure–property relationships, and examples of applications for seven important classes of photoswitches. The authors hope this review serves as a guide and inspiration for researchers approaching the topic, helping them design the most suitable photoswitch for their research and keep the field vibrant and growing (Figure 30).

## Data Availability

Data sharing is not applicable as no new data was generated or analyzed in this study.
